# Identifying SETBP1 haploinsufficiency molecular pathways to improve patient diagnosis using induced pluripotent stem cells and neural disease modelling

**DOI:** 10.1186/s13229-024-00625-1

**Published:** 2024-09-30

**Authors:** Nicole C. Shaw, Kevin Chen, Kathryn O. Farley, Mitchell Hedges, Catherine Forbes, Gareth Baynam, Timo Lassmann, Vanessa S. Fear

**Affiliations:** 1 The Kids Research Institute of Australia, The University of Western Australia, Nedlands, WA Australia; 2 Rare Care Centre, Perth Children’s Hospital, Nedlands, WA Australia

**Keywords:** SETBP1 haploinsufficiency disorder, iPSC, Neural cell modelling, CRISPR, Variants of unknown significance, Neurodevelopmental disorders

## Abstract

**Background:**

SETBP1 Haploinsufficiency Disorder (SETBP1-HD) is characterised by mild to moderate intellectual disability, speech and language impairment, mild motor developmental delay, behavioural issues, hypotonia, mild facial dysmorphisms, and vision impairment. Despite a clear link between SETBP1 mutations and neurodevelopmental disorders the precise role of SETBP1 in neural development remains elusive. We investigate the functional effects of three SETBP1 genetic variants including two pathogenic mutations p.Glu545Ter and SETBP1 p.Tyr1066Ter, resulting in removal of SKI and/or SET domains, and a point mutation p.Thr1387Met in the SET domain.

**Methods:**

Genetic variants were introduced into induced pluripotent stem cells (iPSCs) and subsequently differentiated into neurons to model the disease. We measured changes in cellular differentiation, SETBP1 protein localisation, and gene expression changes.

**Results:**

The data indicated a change in the WNT pathway, RNA polymerase II pathway and identified GATA2 as a central transcription factor in disease perturbation. In addition, the genetic variants altered the expression of gene sets related to neural forebrain development matching characteristics typical of the SETBP1-HD phenotype.

**Limitations:**

The study investigates changes in cellular function in differentiation of iPSC to neural progenitor cells as a human model of SETBP1 HD disorder. Future studies may provide additional information relevant to disease on further neural cell specification, to derive mature neurons, neural forebrain cells, or brain organoids.

**Conclusions:**

We developed a human SETBP1-HD model and identified perturbations to the WNT and POL2RA pathway, genes regulated by GATA2. Strikingly neural cells for both the SETBP1 truncation mutations and the single nucleotide variant displayed a SETBP1-HD-like phenotype.

**Supplementary Information:**

The online version contains supplementary material available at 10.1186/s13229-024-00625-1.

## Background

SET binding protein 1 (SETBP1) haploinsufficiency disorder (SETBP1-HD), otherwise known as intellectual developmental disorder, autosomal dominant 29 (OMIM #616078), is characterised by mild to moderate intellectual disability, speech and language impairment, mild motor developmental delay, behavioural problems, hypotonia, mild facial dysmorphisms and vision impairment [1, 2]. SETBP1-HD is caused by de novo* SETBP1* mutations that cause haploinsufficiency through heterozygous loss-of-function. In contrast, SETBP1 gain-of-function genetic mutations result in a distinct clinical phenotype known as Schinzel-Giedion syndrome, a multi-system disorder characterised by severe neurological and structural abnormalities, which is typically lethal within the first decade of life [3]. While there is a clear association between mutations in *SETBP1* and neurodevelopmental disorders, the role of SETBP1 in brain development is largely unknown.

Clinical findings present in most SETBP1-HD individuals include: motor developmental delay (97%); developmental delay/mild-to-severe intellectual disability; learning difficulties; speech and language disorder (including childhood apraxia of speech). Other variable indications in infants or children include generalized hypotonia of infancy; feeding difficulties; seizures/epilepsy; behaviour consistent with attention-deficit/hyperactivity disorder; anxiety; aggression; sleep disturbances; self-injury; autism spectrum disorder; ophthalmology abnormalities (hypermetropia, myopia, astigmatism, strabismus); digestive problems; and subtle dysmorphic features (ptosis, blepharophimosis, broad nasal bridge, hypertelorism, full nasal tip, and a high arched palate) [1]. The presentation of SETBP1-HD is therefore diverse and determining disease pathogenicity of novel genetic variants is challenging.

SETBP1 is a ~ 170 kDa protein that is expressed in most tissues. It is predicted to stabilise its binding partner, SET, and mediate molecular processes such as histone acetylation, phosphatase activity, DNA repair and cell cycle control through this interaction. Additionally, other proposed roles for SETBP1, independent of SET, include DNA binding and activity as a transcription factor and epigenetic regulator [4]. Interestingly, loss of function mutations in *SET* also lead to a clinical phenotype including intellectual disability, developmental and speech delay and motor impairment [5, 6], similar to SETBP1-HD. While this suggests that the interaction between SETBP1 and SET is key in the pathogenesis of SETBP1-HD, there remains a paucity of data regarding the molecular mechanisms that underlie disease development.

As with many rare diseases, there remains a challenge in the diagnosis of SETBP1-HD as patients often present with symptoms varying in severity, some of which are common to a variety of other genetic neurodevelopmental disorders. Genetic variants can be identified by genomic sequencing. However, unless previously observed, variants are frequently classified as a variant of unknown significance (VUS). These variants require validation in specialised laboratories, which creates a major delay in disease diagnosis. At present, 562 of the 1444 single-gene SETBP1 variants in the ClinVar database are classified as VUS (accessed 03 April 2024) representing a significant bottleneck in patients receiving a definitive diagnosis. Accordingly, new approaches are required to both determine SETBP1-HD disease mechanisms and interpret the effect of SETBP1 VUS on cellular function and disease.

In the current study, we created an isogenic iPSC model of SETBP1-HD using CRISPR/Cas9 genome editing technology to determine the effect of two truncation, loss-of-function *SETBP1* variants SETBP1 p.Glu545Ter and SETBP1 p.Tyr1066Ter, herein termed PATH2 and PATH3, respectively. In addition, we derived iPSCs which harbour a single nucleotide variant in the SET domain, SETBP1 p.Thr1387Met, herein termed VUS2. iPSC disease modelling to neural cells was used to determine the effect on molecular pathways during neural cell differentiation. The study identifies changes in the molecular pathways that drive SETBP1-HD to improve disease knowledge and better inform patient diagnosis and treatment.

## Methods

### IPSC culture

KOLF2-C1 (KOLF2; HipSci) cells were maintained on Matrigel (hESC-Qualified, LDEV-Free; Corning)-coated tissue culture treated plates in TeSR-E8 media (STEMCELL technologies). KOLF2 cells were passaged using Gentle Cell Dissociation Reagent (GCDR; STEMCELL Technologies) according to the manufacturer’s instructions. KOLF2 cells were cryopreserved in KnockOut™ Serum Replacement medium (Gibco) supplemented with 10% DMSO. Upon thaw, TeSR-E8 media was supplemented with 10 µM ROCK Inhibitor (Y-27632, STEMCELL Technologies) for 24 h after plating. All iPSC cultures were maintained at 37 °C, 5% CO_2_, unless otherwise stated.

### CRISPR/Cas9 SETBP1 single nucleotide variant (SNV) transfection

Two pathogenic and one VUS SETPB1 SNVs were selected from the ClinVar database: PATH2 (c.1663G > T; p.Glu545Ter; ClinVar accession VCV000523811.2), PATH3 (c.3198C > A; p.Tyr1066Ter; ClinVar accession VCV000931693.3) and VUS2 (c.4160C > T; p.Thr1387Met; ClinVar accession VCV000523514.7). CRISPR RNA (crRNA) were designed, along with homology directed repair (HDR) templates, for each SNV using the Alt-R HDR Design Tool (Integrated DNA Technologies), the protospacers were: PATH2 SNV ^5′^ACCTAGCACCATGCTTCGAG^3′^, PATH3 SNV ^5′^GGTACTGACCGTAATAACCA^3′^, VUS2 SNV ^5′^TCTGCAGCAACGTCGGATGC^3′^. Single strand DNA HDR repair strands were Alt-R modified (Supplementary Table 1).

KOLF2 cells grown to 60% confluence, dissociated with GCDR and seeded at 2.5 × 10^5^ cells in 400 µL TESR-E8/well supplemented with 10 µM ROCK Inhibitor in Matrigel-coated 24-well plates. Single guide RNA (sgRNA) was prepared in Duplex Buffer (IDT) with 1 µM crRNA and 1 µM AltR CRISPR-Cas9 tracrRNA ATTO (IDT), incubated 5 min at 95 °C and cooled to room temperature. sgRNA was diluted to 10 nM in OPTIMEM (Gibco) with 10 nM AltR s.p.HiFi Cas9 Nuclease V3 (IDT), incubated for 10 min at room temperature and ssDNA HDR template added to a final concentration of 10 nM. Cells were transfected with Lipofectamine as previous [7, 8] with AltR HDR enhancer V2 (IDT) at a final concentration of 1 µM, cells incubated for 48 h at 32 °C with media change at 24 h to remove ROCK Inhibitor. Subsequently, cells were maintained at 37 °C until confluent and expanded to prepare vials for cryopreservation and gDNA extraction (PureLink™ Genomic DNA Mini Kit, Life Technologies) for amplicon sequencing as previous [7].

### Amplicon sequencing

Next-generation amplicon sequencing was performed using the MiniSeq Sequencing System (Illumina ©) as previously described [9, 10]. In brief, PCR amplicon products corresponding to the SNV sites in *SETBP1*, PATH2, PATH3 and VUS2, were prepared from extracted gDNA with forward and reverse amplicon primers (Supplementary Table 2) and Phusion High-Fidelity PCR Master Mix with HF Buffer (Thermo Scientific). Purified samples were barcoded with TruSeq primers (IDT) and *SETBP1* sequences determined with paired-end, 250 bp DNA amplicon sequencing with a MiniSeq Mid-Output Kit, 300 cycles. Reads were aligned to the WT or HDR amplicon with CRISPResso2 software [11] to determine variant representation in each sample.

### Cloning, CRISPR off-target analysis and karyotyping

Clonal iPSC lines were derived from transfected iPSC cultures. Briefly, iPSCs were dissociated into single cells and seeded at low density in Matrigel-coated 6-well tissue culture plates. Individual colonies were manually picked under sterile conditions, clonally expanded, and screened by amplicon sequencing. A second round of cloning and genetic screening was performed to ensure a pure clonal cell population.

The top six off-target CRISPR crRNA sites were confirmed as WT following PCR amplification of 380-680 bp products and Sanger sequencing (AGRF, WA; Supplementary Table 3). Further, the 250 bp product from amplicon sequencing was Sanger sequenced for each SETBP1 clone (Supplementary Fig. 1A; 10.5281/zenodo.13756482 lists all Sanger sequencing files). Genomic DNA samples from SETBP1 iPSC lines were screened for 8 common karyotypic abnormalities reported in human embryonic stem cells and iPSCs using the hPSC Genetic Analysis Kit (STEMCELL technologies) according to the manufacturer’s instructions. Briefly, real-time quantitative PCR was performed in triplicate for each DNA sample and primer/probe combination using the Quantstudio™ Flex system (Applied Biosystems) at cycling conditions specified by the hPSC Genetic Analysis kit instructions. Threshold cycle (Ct) was determined using the Quantstudio™ software and uploaded to the online analysis application (www.stemcell.com/geneticanalysisapp) where Ct values were normalised to the kit’s endogenous control and standardised to a validated diploid control for the regions analysed. Calculated copy numbers across all loci were compared using a one-way ANOVA with a Tukey post-hoc test where copy numbers < 1.8 or > 2.2 with a *p* value of < 0.05 indicated the presence of a karyotypic abnormality in the culture.

### Neural progenitor cell differentiation

Differentiation of PATH2, PATH3, VUS2 and WT SETBP1 iPSC clones (Supplementary Table 23) into neural progenitor cells (NPCs) were induced using the STEMdiff SMADi Neural Induction Kit (STEMCELL technologies). At day 0, 6, 12, 18 and 24 of the differentiation cells were assessed for expression of pluripotency (OCT3) and neural cell (PAX6, NESTIN) markers by flow cytometry. Briefly, cells were harvested using Accutase (STEMCell Technologies), stained for viability using FVS780 (Becton Dickinson), fixed and permeabilised with the eBoiscience™ FOXP3/Transcription Factor Staining Buffer (Thermo Fisher Scientific) according to the manufacturer’s instructions. Cells were then incubated for 30 min at room temperature with OCT3-AF488 (40/Oct-3; BD), PAX6-PE (O18-1330; BD) and Nestin-AF647 (10C2; BioLegend) primary antibodies and analysed on a LSRFortessa (Becton Dickinson). The cell gate was set using FSC and SSC followed by gating for single cells and live cells. Frequency of protein marker expression was expressed as a percentage of live cells. Statistical analysis was performed with two-way ANOVA and Tukey’s multiple comparison test using GraphPad Prism. Additionally, cells were collected for RNA extraction using the RNeasy Minikit (Thermo Fisher) with DNase on column treatment at day 0 and day 24 of the differentiation experiment.

### SETBP1 immunohistochemistry

PATH2, PATH3, VUS2 and WT SETBP1 iPSC clones and NPCs were cultured on Matrigel-coated chamber slides (ibidi). Cells were fixed with 3.7% formaldehyde (Sigma-Aldrich) for 20 min at room temperature and washed with DPBS. Cells were subsequently permeabilised with 0.1% Triton-X-100 for 15 min and blocked with Intercept® Blocking Buffer (LI-COR) for 1 h at room temperature. Cells were incubated overnight at 4 °C with rabbit polyclonal SETBP1 antibody (1:100; Invitrogen) or NESTIN-AF594 (1:100; C2; BioLegend) and DCX (1:25; Invitrogen). Cells were washed with 0.05% Tween-20 (Sigma-Aldrich) and treated with Alexa-Fluor 488-conguated anti-rabbit antibody (1:1000; Invitrogen) for 1 h at room temperature. Residual secondary antibody was removed, and nuclei stained using Nucblue stain (Invitrogen) according to the manufacturer’s instructions. Antibody staining was visualised using a Nikon Eclipse TS2R inverted fluorescence microscope, images captured using a monochrome DS-Qi2 camera (Nikon) and immunofluorescence images processed using NIS-Elements software (v.5.21.00) and Adobe Photoshop (v.22.3.1) to add colour and merge channels. A negative control containing no primary antibody was used to set the background fluorescence.

### RNA sequencing

RNA integrity was determined using the Agilent TapeStation system (Genomics WA). RNA sequencing was performed according to SureSelect Strand-Specific RNA Library preparation for Illumina Multiplexed sequencing, paired-end, 100 bp, 30 M read RNA sequencing on the NOVAseq 6000 platform (Illumina, USA) at Genomics WA (Perth, Australia).

### Pre-processing, exploratory data analysis and differential analysis

Processing of raw sequencing reads, differential gene expression analysis, and enrichment analyses were performed as previously described [8] with the following modifications: normalized log2 counts per million data were calculated using a prior count of 0.5. Enrichment analyses were performed using GSEA functions embedded in the clusterProfiler (v.4.8.2) [12] and DOSE (v.3.26.1) [13] packages, querying against the DisGeNET, DO and GO databases. The full analysis script is reported in Supplemental Experimental Procedures SETBP1_Analysis Script.

### Comparison to publicly available RNAseq data

RNAseq data for wild-type NPCs were extracted from Version 2.1 of ARCHS4 human gene level expression data using h5read [14, 15]. Genes were filtered, limma-trend models fit, and PCA plot generated as previous [8].

## Results

### Generating iPSC clones with heterozygous genotypes for PATH2, PATH3 and VUS SETBP1 variants

**Fig. 1:**
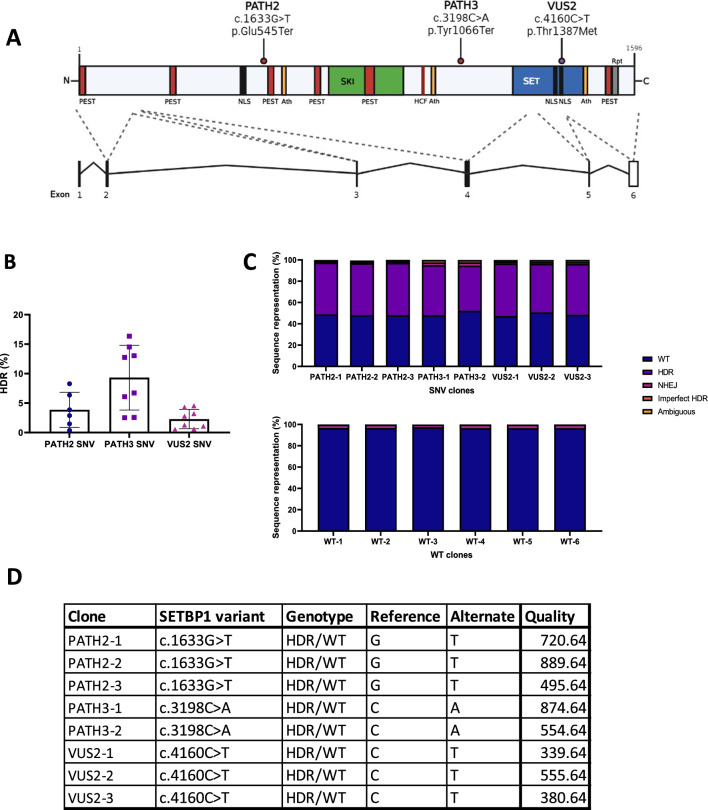
Generation of iPSC clones harbouring SETBP1 variants using CRISPR/Cas9 gene editing. **A** Schematic representation of the SETBP1 protein indicating the PATH2, PATH3 and VUS2 variants introduced into the KOLF2 iPSC genome using CRISPR/Cas9 gene editing. Five exons encode isoform A (1596 amino acid residues) of the SETBP1 protein. Truncating variants PATH2 and PATH3 are not located in any functional domains, however the missense variant, VUS2, is in an NLS region within the SET-binding domain. PEST: proline, glutamic acid, serine, and threonine rich sequence; NLS: nuclear localisation signal; Ath: AT hook; SKI: SKI homology region; HCF: HCF-1 binding motif; SET: SET-binding domain; Rpt: repeat. **B** CRISPR/Cas9 HDR efficiency for SETBP1 SNV integration into iPSC genomes. Data presented as mean ± s.d. HDR: homology directed repair; SNV: single nucleotide variant. **C** SETBP1 SNV and WT derived cell clones. Bar graphs show genotype for three heterozygous PATH2 variant cell lines, two heterozygous PATH3 variant cell lines, three heterozygous VUS2 variant cell lines, and the six wild-type clones selected as isogenic controls. SNV: single nucleotide variant; WT: wild-type; NHEJ: non-homologous end-joining. **D** Table of variant calling metrics for SETBP1 in NPC samples. Quality: Phred-scaled quality score

Independent CRISPR/Cas9 HDR transfections introduced the three *SETBP1* SNVs into the KOLF2 iPSCs (Fig. 1A). HDR efficiency varied across the PATH2 SNV (3.86 ± 2.98%; n = 6), PATH3 SNV (9.32 ± 5.49%; n = 8) and VUS2 SNV (2.28 ± 1.64%; n = 8) transfections (Fig. 1B). Single cell clones were derived from the transfections with maximal HDR efficiency for PATH2 (8.37%) and PATH3 (16.36%); and VUS2 (4.31%, 4.54% and 3.21%). Derived cell clones were assessed with SETBP1 amplicon sequencing on genomic DNA (minimum of 30,000 reads), and analysed with CRISPResso2 software [11]. Three heterozygous (HDR/WT) clones for the PATH2 SNV and VUS2 SNV, and two heterozygous clones for the PATH3 SNV were generated (Fig. 1C). A further, six homozygous wild-type (WT/WT) clones were selected as isogenic controls.

To confirm the presence of the variants, we applied the standard Genome Analysis Toolkit (GATK) best practices pipeline to call variants in our RNAseq data [16]. We were able to reliably detect all variants in our differentiated neural progenitor cells with high confidence (Phred-scaled probability that the site has no variant less than 10^−33^ for all variants). Data indicates the expression of both the normal wild-type and expected genetic variant transcript in each cell line (Fig. 1D, and Supplementary Fig. 1).

Next, clones were assessed at the genomic DNA level for chromosomal abnormalities by qPCR and were similar to parental KOLF2 iPSCs (Supplementary Fig. 2). Next, we examined the top six off-target sites for each SETBP1 crRNA using Sanger Sequencing (Supplementary Figs. 3, 4, 5) and determined normal gDNA integrity. All iPSC clones maintained a stem cell-like morphology during cloning (Supplementary Fig. 6), and the expression of stem cell markers OCT3 and NANOG (Supplementary Fig. 7).

### SETBP1 PATH2, PATH3 and VUS2 genetic variants alter neural cell differentiation

**Fig. 2:**
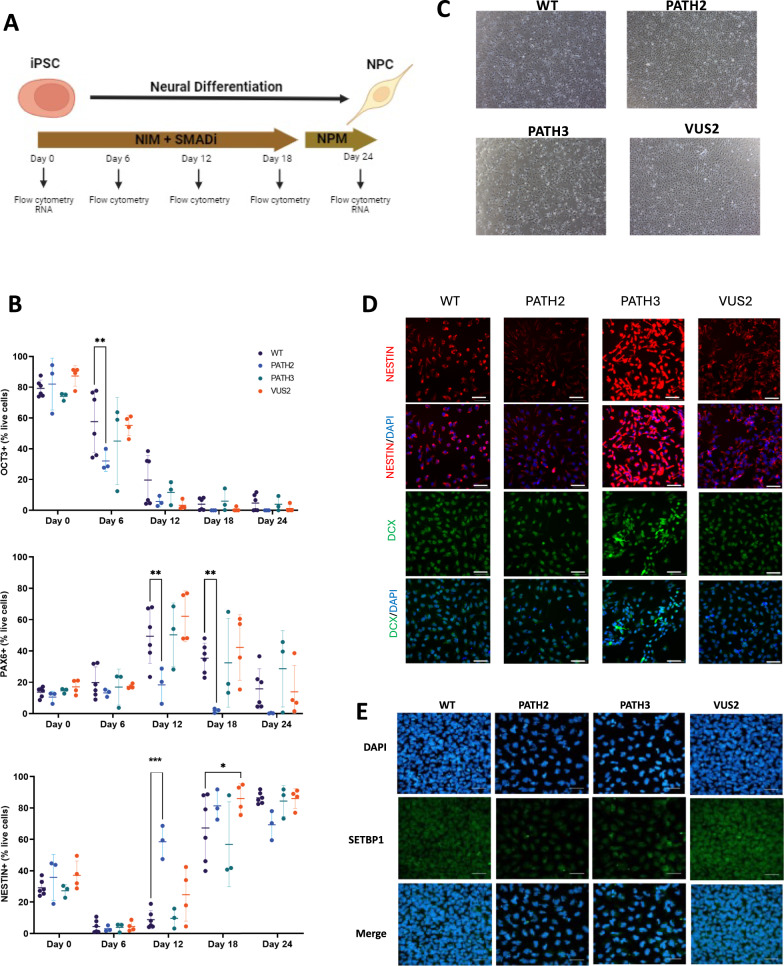
Differentiation of SETBP1 iPSC clones into neural progenitor cells. **A** Neural differentiation schematic showing differentiation of iPSCs into neural progenitor cells (NPCs) using neural induction media (NIM) and SMADi followed by neural progenitor media (NPM). Cells were harvested at selected timepoints across differentiation for pluripotency and neural marker analysis using flow cytometry and RNAseq. Image generated using BioRender. **B** Pluripotency and neural marker expression across neural differentiation. Graph plots indicate OCT3, PAX6 and NESTIN expression was assessed by flow cytometry in differentiating iPSC clones at 6-day intervals. Data presented as mean ± s.d.*p < 0.05; **p < 0.01; ***p < 0.001. WT: wild-type. **C** Morphology of NPCs derived from SETBP1 gene-edited iPSC clones. Representative images of NPCs harbouring PATH2, PATH3, VUS2 SETBP1 variants and WT NPCS at 4X objective magnification. **D** Representative images of neural marker, NESTIN and DCX, immunostaining in NPCs harbouring *SETBP1* variants. Scale bar 50 µm. **E** Representative images of SETBP1 staining (green) relative to nuclear staining (blue) in iPSC-derived NPCs. Scale bar 50 µm

Neural differentiation was induced in each SETBP1 iPSC lines and cells harvested for analysis (Fig. 2A). Cell populations were analysed for pluripotency and neural marker expression after gating on cells, single cells, and then live cells for each timepoint across neural differentiation (Supplementary Fig. 7). Temporal changes in the percentage frequency expression of OCT3, PAX6 and NESTIN expression were determined (Fig. 2B). The proportion of cells expressing OCT3 was highest at day 0 of differentiation for SETBP1 PATH2 (82.03 ± 16.88%; n = 3), PATH3 (74.0 ± 2.43%; n = 3), VUS2 (87.33 ± 6.62%; n = 4) and WT (79.23 ± 5.40%; n = 6) populations. The frequency of OCT3 expression decreased across neural differentiation and there were no significant differences in the proportion of cells expressing OCT3, PAX6, or NESTIN at the iPSC (day 0) or NPC (day 24) stage of differentiation. Interestingly, at day 6 of neural differentiation, the percentage frequency of OCT3^+^ cells in SETBP1 PATH2 was decreased compared to WT (*p* = 0.005). The percentage frequency of PAX6 expression peaked at day 12 for SETBP1 PATH2 (18.41 ± 11.35%; n = 3), PATH3 (50.27 ± 20.42%; n = 3), VUS2 (62.13 ± 16.26%; n = 4) and WT (49.40 ± 17.38; n = 6) cells. Notably, there were significantly decreased PAX6 percentage frequency expression in SETBP1 PATH2 cells at day 12 (*p* = 0.008) and day 18 (*p* = 0.004) compared to the WT. NESTIN expression peaked at day 18 for SETBP1 PATH2 (81.37 ± 9.71%; n = 3) and at day 24 for SETBP1 PATH3 (84.4 ± 9.86%; n = 3), VUS2 (86.05 ± 6.30%; n = 4) and WT (86.42 ± 3.63%; n = 6) cells. There was an increase in the percentage frequency of NESTIN at day 12 in SETBP1 PATH2 cells compared to the WT (*p* < 0.001), and at day 18 in SETBP1 VUS2 cells compared to WT (*p* = 0.044).

Throughout the neural differentiation there was a transition of cellular morphology observed, with large clustered iPSCs at day 0; to small, evenly dispersed, and elongated cells, with evidence of neurites projecting from the cell body, indicative of neural cells at day 24 (Fig. 2C). Next, using immunofluorescence staining we examined neural cell marker and SETBP1 expression. The neural markers NESTIN and DCX were expressed in both WT and SETBP1 genetic variant NPCs, with similar expression in WT, PATH2 and VUS2 cells, with increase expression in the PATH3 cells (Fig. 2D). Finally, in the NPCs, a nuclear pattern of SETBP1 expression was determined using immunofluorescence staining and this was consistent across the different SETBP1 genotypes (Fig. 2D, and Supplementary Fig. 8). Of note visually there appears to be reduced SETBP1 nuclear staining, that is more diffuse, in the SETPB1 PATH2 and SETBP1 PATH3 cells, when compared to the control or VUS2 cells. The SETBP1 PATH2 and PATH3 truncated proteins are likely subject to non-sense mediated decay, or as they may not localise to the nucleus efficiently due to the loss of two carboxy terminal NLS. Alternatively, the SETBP1 VUS2 is a missense mutation that may be stably expressed, and it retains all three NLS signals.

These data indicate temporal differences in neural progenitor cell differentiation from iPSCs harbouring SETBP1 PATH2 and SETBP1 VUS2 variants compared to the healthy matched control cells.

### Transcriptomic data supported neural identity of differentiated iPSCs

**Fig. 3:**
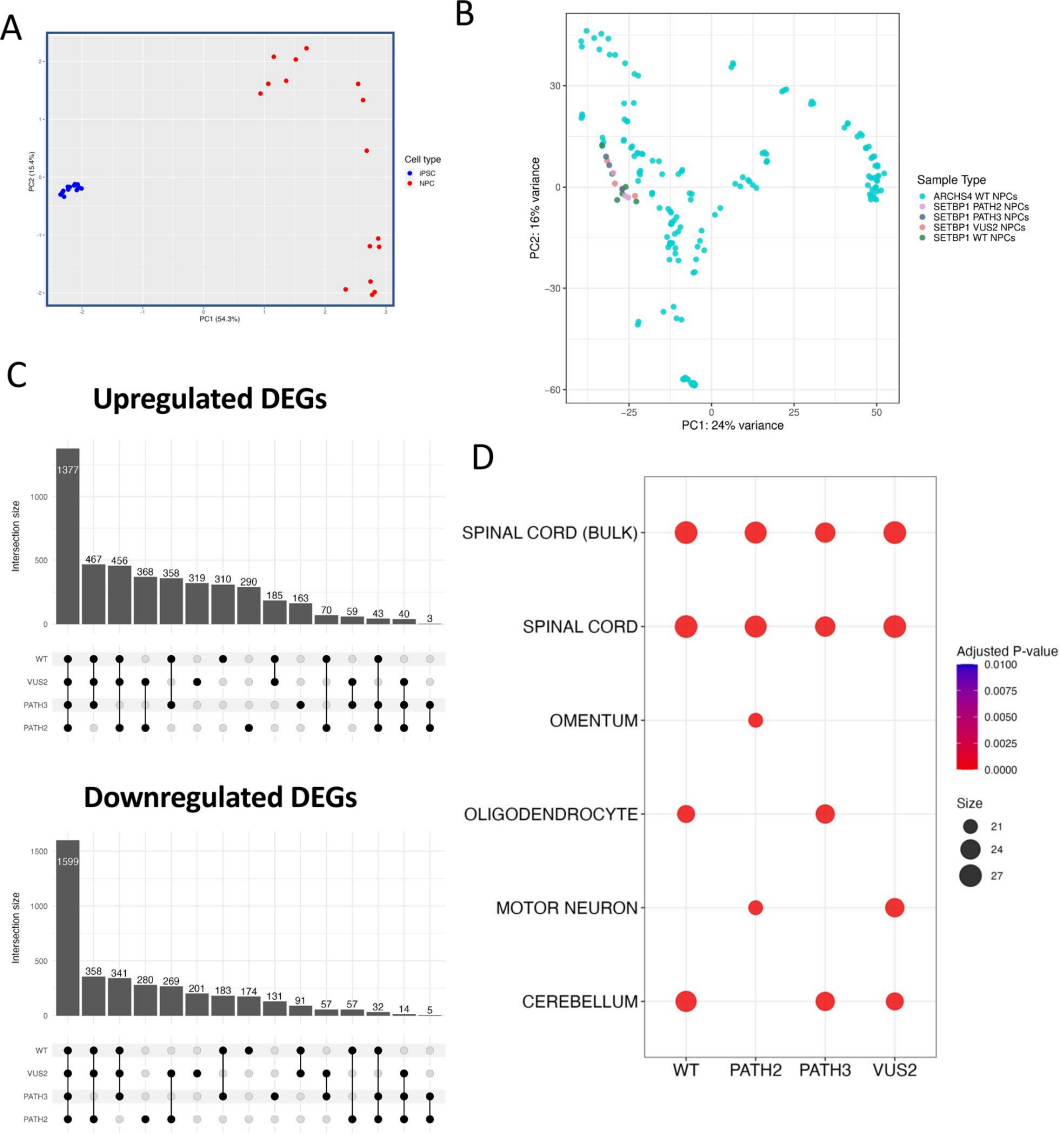
Neural identity of differentiated SETBP1 iPSC clones. **A** PCA plot demonstrating clear separation between iPSC and NPC samples based on gene expression. **B** PCA plot comparing SETBP1 experimentally derived NPCs with wild-type NPCs from the ARCHS4 database. **C** Numbers of DEGs up and downregulated in NPCs defined by *SETBP1* genotype. **D** Analysis of top 50 significant upregulated genes in NPCs compared to iPSCs using Enrichr demonstrated enrichment of neural tissue gene sets in the ARCHS4 tissue database

Next, we performed transcriptomics analysis to determine changes in gene expression and molecular and cellular pathways during neural cell differentiation. Principal component analysis (PCA) indicated clear separation between iPSCs and NPCs (Fig. 3A). To confirm derivation of NPCs from SETBP1 genetic variant and WT iPSCs, we integrated our transcriptomic data with publicly available data in the ARCHS4 database, and demonstrate that the SETBP1 WT, SETBP1 PATH2, SETBP1 PATH3, and SETBP1 VUS NPCs grouped with wild-type NPCs using PCA (Fig. 3B). Both experimentally derived and ARCHS4 NPCs align closely along principal components one and two, indicating a high degree of similarity.

There were 6101 differentially expressed genes (DEGs) between iPSCs and NPCs for SETBP1 WT cells, 5261 DEGS for SETBP1 PATH2 cells, 4872 DEGS for SETBP1 PATH3 cells, and 6201 DEGS for VUS2 cells, including up and down regulated genes (Fig. 3C; Supplementary Tables 4, 5, 6). Notably, at the transcript level there was down regulation of OCT3 and NANOG, and upregulation of neural transcripts including NES, MAP2, DCX, POU3F3, NR2F1, NR2F2, and SOX5 (Supplementary Fig. 9).

Analysis using EnrichR software and the top 50 upregulated genes during neural differentiation demonstrated enrichment of gene sets associated with neural tissues (Fig. 3D). The top four ARCHS4_tissues gene sets enriched during neural differentiation for SETBP1 WT cells were spinal cord (*p* = 1.75 × 10^−11^), spinal cord-bulk (*p* = 1.75 × 10^−11^), cerebellum (*p* = 8.28 × 10^−10^) and oligodendrocytes (*p* = 2.07 × 10^−7^). For SETBP1 PATH2 cells the top enriched gene sets were spinal cord (*p* = 1.55 × 10^−10^), spinal cord-bulk (*p* = 1.55 × 10^−10^), motor neuron (*p* = 7.07 × 10^−7^) and omentum (*p* = 7.07 × 10^−7^) while for SETBP1 PATH3 differentiation they were spinal cord (*p* = 9.67 × 10^−9^), spinal cord-bulk (*p* = 9.67 × 10^−9^), cerebellum (*p* = 3.35 × 10^−8^) and oligodendrocyte (*p* = 3.35 × 10^−8^). The top gene sets enriched for SETBP1 VUS2 cells across neural differentiation were spinal cord (*p* = 1.78 × 10^−11^), spinal cord-bulk (*p* = 1.78 × 10^−11^), motor neuron (*p* = 4.38 × 10^−8^) and cerebellum (*p* = 2.11 × 10^−7^). These data confirmed the differentiation of iPSCs into neural cells for each SETBP1 genotype consistent with findings observed for neural cell marker expression and cell morphology.

### Disease pathways in SETBP1 genetic variant NPCs

To determine whether the SETBP1 genetic variant NPCs displayed a SETBP1-HD-like disease phenotype, we performed gene set enrichment analysis (GSEA) and identified DisGeNET and Disease Ontology (DO) gene sets enriched in SETBP1 variant NPCs compared to WT NPCs.

**Fig. 4:**
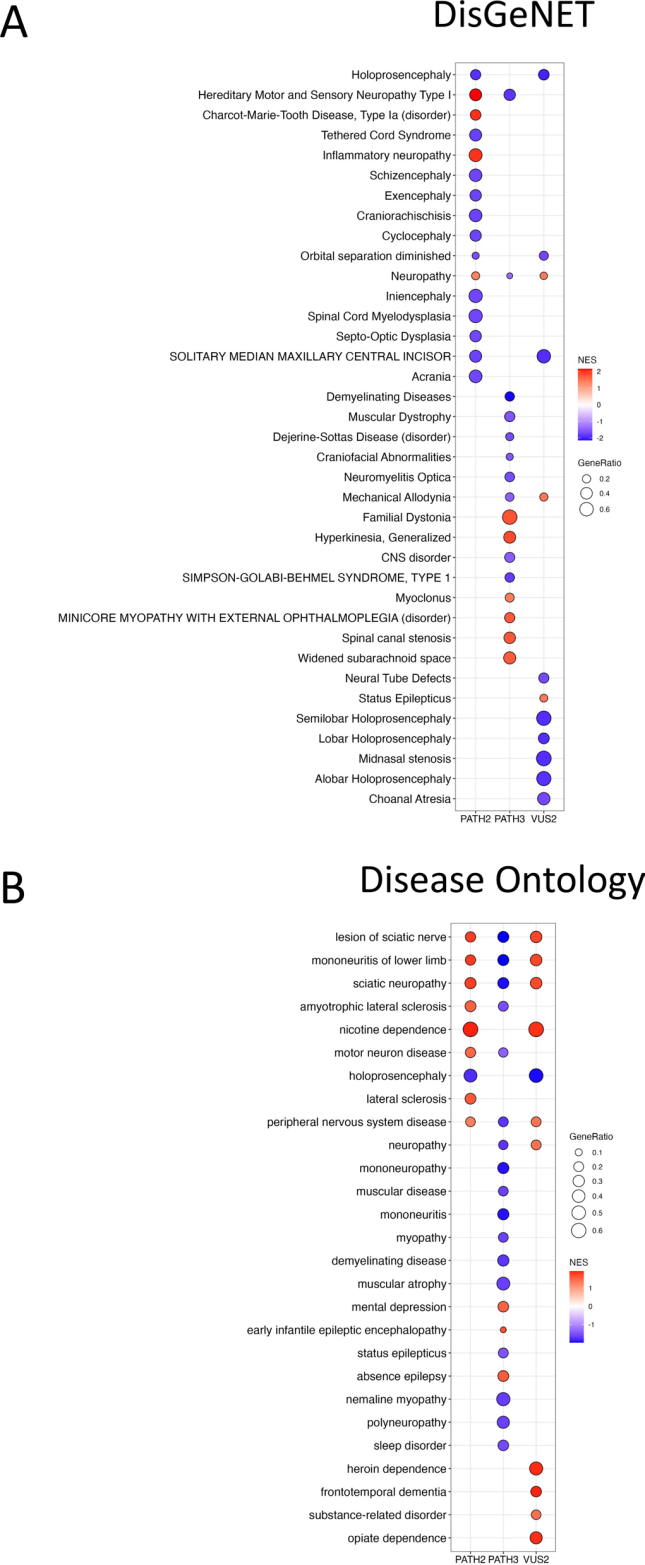
Disease associated gene sets that are significantly dysregulated in SETBP1 variant NPCs compared to WT NPCs. Selected gene set terms that are significantly dysregulated in SETBP1 PATH2, SETBP1 PATH3 and SETBP1 VUS2 NPCs for DisGeNET (**A**) and Disease Ontology (**B**) databases. Full list of significant terms for each SETBP1 SNV is available in the supplementary data

In DisGeNET, 67 gene sets were significantly dysregulated in SETBP1 PATH2 NPCs in comparison to WT NPCs (Supplementary Table 7). Gene sets related to nervous system disorders were significantly altered in genetic variant NPCs compared to WT NPCs (Fig. 4A) and included Hereditary Motor and Sensory Neuropathy Type 1; Charcot-Marie-Tooth Disease, Type Ia; and Holoprosencephaly, amongst others. In SETBP1 PATH3 NPCs compared to WT NPCs 655 gene sets were significantly dysregulated (Supplementary Table 8) including: Hereditary Motor and Sensory Neuropathy Type 1; Familial Dystonia; widened subarachnoid space; minicore myopathy with external ophthalmoplegia (disorder); and Generalized Hyperkinesia. In the SETBP1 VUS2 NPCs compared to WT NPCs, 57 gene sets were significantly dysregulated in the DisGeNET database (Supplementary Table 9). Similar to the SETBP1 PATH2 and PATH3 NPCs the VUS2 NPCs significantly dysregulated gene sets relating to nervous system disorders including Neuropathy; Holoprosencephaly; Orbital separation diminished; and mechanical allodynia. Other significantly dysregulated pathways in VUS2 NPCs included: Status Epilepticus; Neural Tube Defects; Midnasal Stenosis; and choanal atresia.

In addition, we performed analysis using disease ontology (DO; Fig. 4B) database. The SETBP1 PATH2 NPCs had 23 gene sets significantly dysregulated compared to WT NPCs (Supplementary Table 10) and included sciatic neuropathy; motor neuron disease; holoprosencephaly; and peripheral nervous system disorder. In comparison to WT NPCs, SETBP1 PATH3 NPCs had a total of 367 DO gene sets dysregulated (Supplementary Table 11) including mononeuritis of lower limb; early infantile epileptic encephalopathy; muscular disease; and status epilepticus. The SETBP1 VUS2 NPCs had a total of 29 DO gene sets that were significantly dysregulated in comparison to WT NPCS (Supplementary Table 12). In SETBP1 VUS2 NPCs, DO pathway terms in common with SETBP1 PATH2 and SETBP1 PATH3 NPCs included lesion of sciatic nerve; mononeuritis of lower limb; sciatic neuropathy; and peripheral nervous system disease. DO terms unique to SETBP1 VUS2 NPCs included frontotemporal dementia; and substance related disorder.

This data identified many terms indicative of a SETBP1-HD-like phenotype. Holoprosencephaly is caused by incomplete separation of the prosencephalon (embryonic forebrain) to divide into double lobes of the cerebral hemispheres. Widened subarachnoid space is associated with motor and language delay. Other neural symptoms consistent with SETBP1-HD phenotype include hyperkinesia in ADHD; sensory changes in autism; dysmorphic facial features, ophthalmological abnormalities such as retinopathy; and alterations in digestion related pathways. Notably, a number of dysregulated pathways identified in the pathogenic truncation mutations, were also identified for the novel single nucleotide variant SETBP1 VUS2. However, there was a higher degree of similarity in the direction of enrichment score between SETBP1 VUS2 and SETBP1 PATH2 NPCs compared to SETBP1 PATH3 NPCs.

### Variants in SETBP1 cause a specific perturbation to the transcriptome

**Fig. 5:**
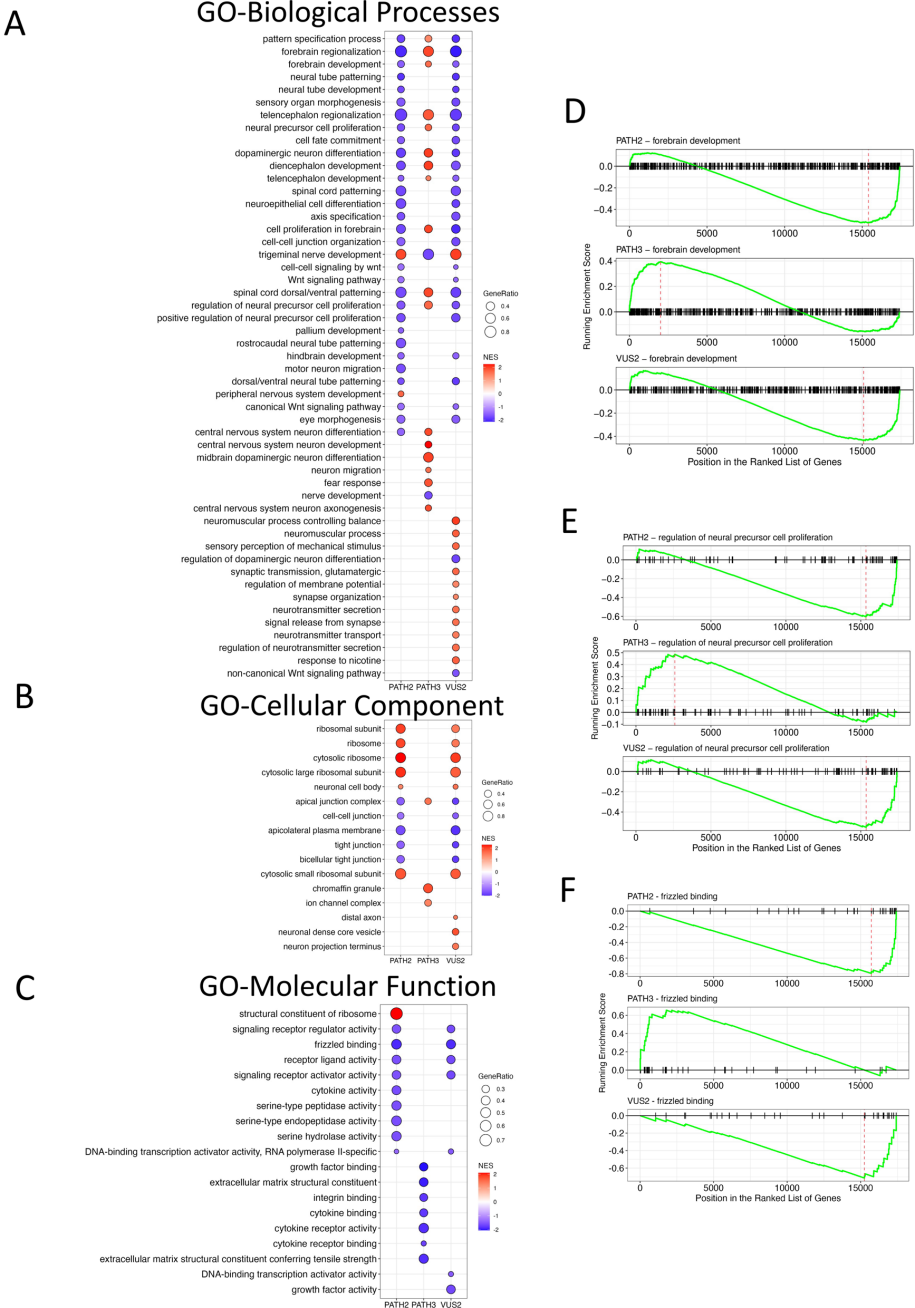
Gene sets using Gene Ontology terms that are significantly dysregulated in SETBP1 variant NPCs compared to WT NPCs. Selected gene set terms that are significantly dysregulated in SETBP1 PATH2, SETBP1 PATH3 and SETBP1 VUS2 NPCs for Gene Ontology (GO)-Biological Process (**A**), GO-Cellular Component (**B**), and (**C**) GO-Molecular Function terms. GSEA plots demonstrate opposing patterns of gene set expression between PATH2 and VUS2 compared to PATH3 for GO-BP terms forebrain development (**D**) and regulation of neural precursor cell proliferation (**E**). Likewise, gene set expression for GO-MF term frizzled binding (**F**) was aligned for PATH2 and VUS2 and contrasted with PATH3. Full list of significant terms for each SETBP1 SNV is available in the supplementary data

To further investigate changes in cellular pathways we examined differential gene expression (DEG) and performed GSEA. SETBP1 PATH2, SETBP1 PATH3 and SETBP1 VUS2 NPCs were compared to WT controls to determine significant pathway gene set changes in Gene Ontology (GO)-Biological Process (Fig. 5A), GO Cellular Component (Fig. 5B), and GO Molecular Function (Fig. 5C).

In initial gene expression analysis, the SETBP1 PATH2 variant had 642 DEGs in comparison to WT NPCs, including 332 genes that were upregulated and 310 genes that were downregulated (Supplementary Table 13). In contrast, SETBP1 PATH3 NPCs had no DEGs compared to WT, while a single gene was significantly differentially expressed in SETBP1 VUS2 NPCs compared to WT NPCs: TRIM4 (logFC: − 3.86; *p* = 6.23 × 10^−7^) in comparison to WT NPCs. Next, as there were temporal changes in neural markers during NPC differentiation, we determined the DEG for the difference of differentiation, comparing the changes in SETBP1 PATH2 differentiation to SETBP1 WT differentiation. There were 32 significant DEGs (Supplementary Table 13), and the top upregulated gene was ISL2 a negative regulator of neuron differentiation.

In GO Biological Process (GO-BP) terms, comparison of genetic variant SETBP1-like NPCs to WT NPCs demonstrated significant pathway dysregulation (Supplementary Tables 14, 15, 16) in neural networks associated with forebrain development; neural cell specification; and nervous system development. GO-BP significant gene sets common to SETBP1 PATH2, PATH3 and VUS2 NPCs in comparison to WT cells included: pattern specification process; forebrain regionalization; cell proliferation in forebrain; trigeminal nerve development; forebrain development; dopaminergic neuron differentiation; regulation of neural precursor cell proliferation; telencephalon development; spinal cord dorsal/ventral patterning; diencephalon development; and central nervous system neuron differentiation. Interestingly, the direction of enrichment scores in forebrain development (Fig. 5D) and regulation of neural precursor cell proliferation (Fig. 5E) for each SETBP1 genotype indicate similarity between SETPB1 PATH2 and SETBP1 VUS2 NPCs.

**Fig. 6:**
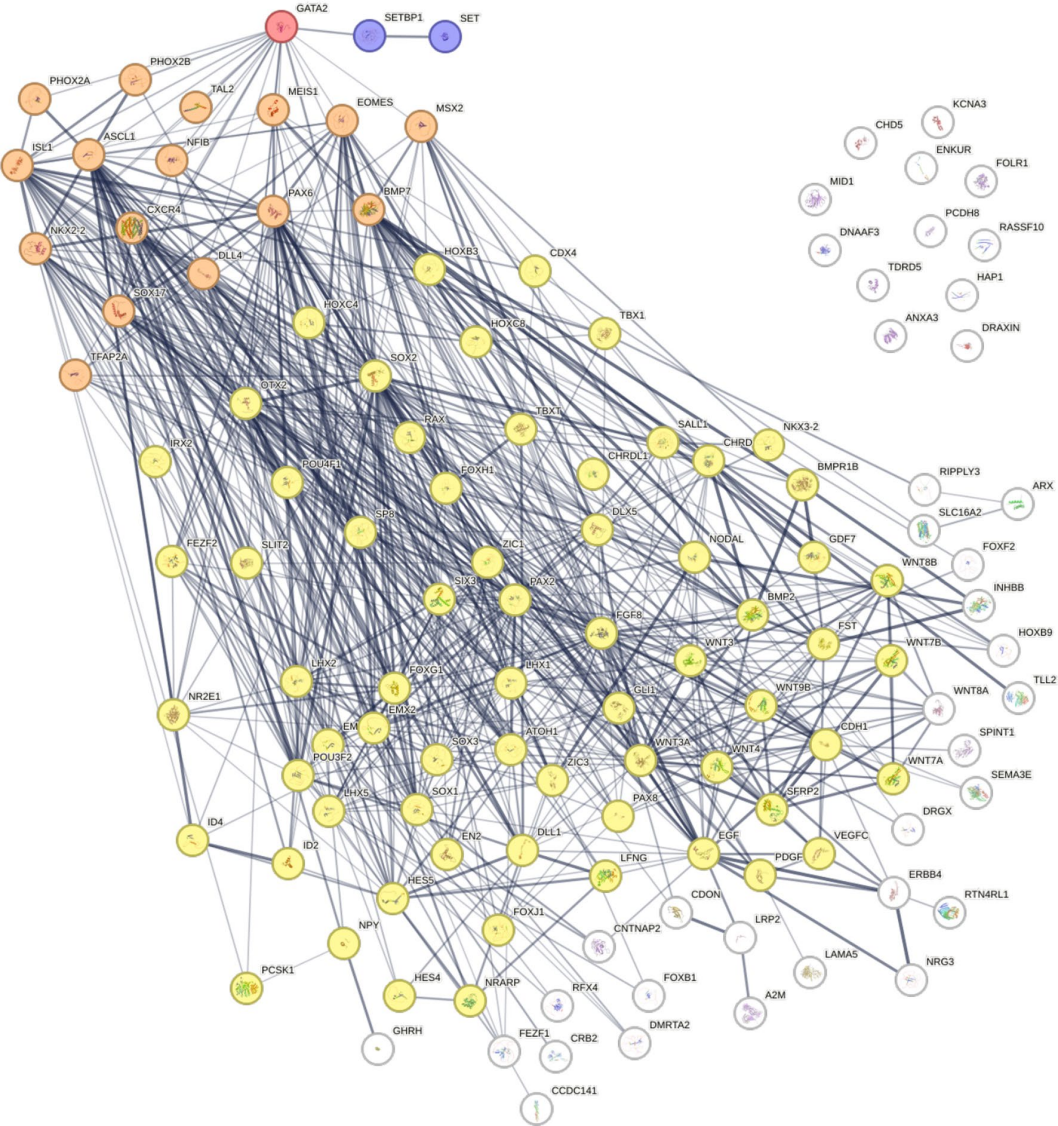
STRING network demonstrating protein associations in dysregulated GO-BP gene sets across the SETBP1 variant NPCs. GATA2 was identified as a key transcription factor linking to SETBP1 and many of the core enrichment genes in dysregulated gene sets in SETBP1 variant NPCs. Proteins directly associated with GATA2 are shown in orange, while proteins indirectly associated with GATA2 are shown in yellow. Level of confidence in association between proteins is represented by the thickness of the line

In further analysis of GO-BP gene pathways, we investigated genes from all common pathways between SETBP1 variants using STRING and identified a central link between SETBP1 pathways genes and GATA2 (Fig. 6). The importance of GATA transcription factors and ability to replace OCT4 to induce pluripotency in somatic cells has been previously reported [17], whilst other research indicates GATA2 has a role in neural specification [18]. Interestingly, in the DEG comparison of SETBP1 PATH2 NPC to iPSC, GATA2 was among the downregulated DEG (logFC =  − 3.4782, adjusted *p* value = 0.0054; Supplementary Table 4).

In GSEA GO-Cellular Component (GO-CC; Fig. 5B), gene sets that were significant for SETBP1 PATH2 NPCs compared to WT (Supplementary Table 17) included neuronal cell body; axoneme; and apical junction complex. In SETBP1 PATH3, significant GO-CC terms (Supplementary Table 18) included apical junction complex; ion channel complex; and transporter complex. In SETBP1 VUS2 NPCs compared to WT, significant pathways were overlapping with SETBP1 PATH2 and SETBP1 PATH3 terms including apical junction complex; neuronal cell body; cell–cell junction; apicolateral plasma membrane; and tight junction. Specific to VUS2 were the gene sets distal axon; neuronal dense core vesicle; and neuron projection terminus (Supplementary Table 19).

The ion channel complex and transporter complex are responsible for selective cell permeability and active transport. Recent findings indicate that these systems co-operate in cell ion homeostasis and can physically interact with neurotransmitter transporters expressed in the brain [19] to form a cellular signalling hub.

In GO Molecular Function (Fig. 5C), gene sets that were significant for SETBP1 PATH2 NPCs compared to WT (Supplementary Table 20) included structural constituent of ribosome; signalling receptor regulator activity; and frizzled binding. The significantly dysregulated gene sets for PATH3 NPCs (Supplementary Table 21) included: growth factor binding; integrin binding; and extracellular matrix structural constituent. In VUS2 NPCs (Supplementary Table 22) pathway gene sets overlapped with PATH2 NPCs including frizzled binding; DNA-binding transcription activator activity, RNA-polymerase II-specific; and signalling receptor regulator activity. The enrichment scores for frizzled binding indicate similarity between PATH2 and VUS2 NPCs (Fig. 5F).

The Frizzled (FZD) pathway is stimulated by WNT ligand binding to the receptor leading to canonical and noncanonical Wnt signalling [23]. Ligands represented in the core enrichment gene sets for both PATH2 and VUS2 NPCs included WNT2B, WNT3/3A, WNT4, WNT7A/B, WNT8A/B and WNT9A/B (Suppl Tables 20 and 22). These factors have a variety of roles in brain development, including patterning and regionalisation [20], neural stem cell self-renewal and differentiation [21], neuronal migration [20], axon guidance and synapse formation [22]. Accordingly, dysfunction of WNT signalling due to SETBP1 mutation likely perturbs neural cell development and function. Components of both the Wnt and the related Hippo Signalling pathways are affected in a SETBP1 variant-dependent manner (Supplementary Fig. 10). In other studies, SETBP1 is identified as a part of a DNA-binding complex that modulates histone methylation to make chromatin more accessible and regulate gene expression. Recent studies propose an epigenetic complex consisting of POLR2A, PHF8/6, MLL1, HCF and SETBP1 that regulates transcription according to histone methylation status at H4K20me and H3K4me [23].

These data indicate that the SETBP1 PATH2, SETBP1 PATH3 and SETBP1 VUS2 NPCs have common GO terms, potentially highlighting overlapping mechanisms of cell dysfunction that may contribute to SETBP1-HD.

## Discussion

The study investigated three genetic variants linked to SETBP1-HD. We determined changes in neural cell differentiation including altered protein expression, transcript levels, and further identified changes in cellular pathways relevant to forebrain development which has been associated with the aetiology of disease. Notably upon examination of pathway changes in the SETBP1-HD like cells we identified relevant terms that related to disease phenotype. In addition, transcriptomics analysis revealed perturbation of the POLR2A DNA binding complex, dysfunction in the WNT/b-catenin signalling, and identified a key role for the transcription factor GATA2. Further, we demonstrate that a SETBP1 missense mutation had overlapping phenotype with pathogenic SETBP1 truncation mutations highlighting that single nucleotide variants in SETBP1 lead to SETBP1-HD-like disease.

The three genetic variants in SETBP1 (p.Glu545Ter, p.Tyr1066Ter and p.Thr1387Met) revealed a spectrum of neural cell phenotypes with overlapping, as well as divergent characteristics. Disease pathways identified were consistent with the core phenotypes characteristic of SETBP1-HD including language delay, autism, ADHD, delayed development, and epilepsy. In addition, the study clearly identified changes in molecular pathways involved in forebrain development in the SETBP1 HD-like NPCs. Recently, others have observed changes in forebrain development in SETBP-HD cellular models [23, 24]. Studies in 293 T cells indicated SETBP1 p.Gly870Ser expression altered cellular pathways relating to forebrain development [23]. In other transcriptomics studies anomalies were identified in forebrain cellular pathways in homozygous SETBP1-KO neural cells [24]. In this study, we investigate genetic variant NPCs that are heterozygous for SETBP1 and therefore demonstrate changes in forebrain cellular pathways that more closely mimic this haplo-insufficiency disease.

The DNA-binding transcription activator activity, RNA-polymerase II-specific pathway was dysregulated in the SETBP1-like neural cells. Recent other studies in cells that stably express the SETBP1 p.G870S mutation, in the SKI domain, indicate that the Ath (AT hooks) of SETBP1 binds DNA and allows activation of gene expression via recruitment of an HCF/KMT2A/pHF8/POLR2A epigenetic complex [23]. Potentially, SETBP1 mutations alter the epigenetic modification of DNA leading to altered gene expression and regulation of cell fate. Notably, *POLR2A* variants have been associated with a recently described neurodevelopmental syndrome with infantile-onset hypotonia and developmental delay with milder phenotypes attributed to haploinsufficiency, and a phenotype that shares overlapping features with SETBP1-HD [25].

SETBP1 knockout models of iPSC to NPC differentiation have demonstrated increased WNT/b-catenin signalling with NPC expansion [24]. The Frizzled binding pathway has cross-regulation with the Hippo and Sonic Hedgehog pathways [26], and is activated by WNT ligands. In keeping with these findings, our findings herein similarly indicate heterozygous SETBP1 genetic variant NPCs with dysregulated Wnt/Beta-catenin signalling.

GATA2 is an important transcription factor that reportedly maintains stem cell pluripotency and later in neural development is involved forebrain development and neural cell specification. Studies indicate that GATA2 and Sonic Hedgehog (SHH) signalling play a role during neuronal differentiation leading to increased yield of serotonergic neurons from embryonic stem cells [27]. Specifically, the SHH-regulated cascade of transcription factors induces GATA2 leading to the generation of rostral (rhomobmere1, r1) serotonergic neurons, which project to the forebrain. Interestingly, studies indicate there is a relationship between GATA2 and SETBP1 with respect to myeloid malignancies, and somatic mutations involving *SETBP1* commonly occur in patients with germline *GATA2* mutations for gene deficiency [28]. However, in the context of neurological disorders, an association between GATA2 and SETBP1 has not been reported. Data in this study indicates a role for GATA2 in driving cellular and molecular pathway perturbations that occur during neural differentiation in SETBP1-HD.

In the majority of SETBP-HD patients, identified pathogenic variants are predominantly truncation mutations leading to gene haploinsufficiency [1]. However as genetic variant detection improves, single nucleotide missense variants are increasingly identified in genetic analysis [29]. The functional consequences of these missense variants in disease require investigation. Here we demonstrate similarity between SETBP1-like neural cells that harbour disease causative truncation mutations, particularly the PATH2 SETBP1 p.Glu545Ter variant, and a missense single nucleotide variant, VUS2 SETBP1 p.Thr1387Met. This study further indicates that there is a spectrum of cellular mechanisms that contribute to the SETBP1-HD disease phenotype that may be *SETBP1* variant-specific, a profile consistent with variations in patient phenotype and presentation [1, 29].

## Limitations

Here we introduce SETBP1 genetic variants into iPSCs and derive neural progenitor cells, however these cells could also be induced to form brain organoids. We noted perturbations to forebrain development in SETBP1-HD like neural progenitor cells, which have capacity for further stimulation of differentiation to form forebrain neurons. Analysis of organoids or mature neural cells may further identify changes in neural cell specification, and perturbations in cellular pathway leading to disease which could be targeted in treatment development.

## Conclusions

The WNT/b-catenin pathway, that is implicated in forebrain development, was disrupted in SETBP1-HD like neural cells, strengthening data for the importance of this region in disease development. In addition, we provide evidence toward a role for GATA2 in perturbation of neural cell differentiation in SETBP1-HD. Importantly, the study indicates that SETBP1 single base gene editing in iPSCs, and neural disease modelling provide a suitable model for human SETBP1-HD. In future studies, we anticipate that induction of forebrain neural cell differentiation, or brain organoids from our SETBP1-HD like cells, will provide further insight into disease mechanism, pathway changes, and gene regulation.

## Supplementary Information

Additional file 1

Additional file 2

Additional file 3

## Data Availability

Raw FASTQ files and processed count data for bulk RNA-seq are available at the Gene Expression Omnibus repository under accession number GSE262710. SETBP1 amplicon sequencing files for selection of clonal cell lines are also available. The supplemental experimental procedures Paper Analysis section provides all the code to reproduce the analysis and figures in this paper.

## Abbreviations

SETBP1: SET Binding Protein 1
SETBP1-HD: SETBP1 haploinsufficiency disorder
HDR: Homologous directed repair
NHEJ: Non-homologous end-joining
WT: Wild type
iPSC: Induced pluripotent stem cells
NPC: Neural progenitor cell
SNV: Single nucleotide variant
NES: Normalised enrichment score
DEG: Differentially expressed gene
GSEA: Gene set enrichment analysis
VUS: Variant of unknown significance
KO: Knock out
GO: Gene ontology
PCA: Principal component analysis
