# Supplementary material for: Identifying SETBP1 haploinsufficiency molecular pathways to improve patient diagnosis using induced pluripotent stem cells and neural disease modelling
Source: Mol Autism. 2024 Sep 30;15:42. doi: 10.1186/s13229-024-00625-1 (PMC11443744; doi:10.1186/s13229-024-00625-1)
Supplement: Supplementary file 1 — Additional file 1 [file 13229_2024_625_MOESM1_ESM.pdf]

SETBP1 Analysis Script

Kevin Chen

February 12, 2024

Contents

1 Preparation **1**

2 Processing DGE object **2**

3 Construction of the PCA plot **5**

4 Differential gene expression analysis **6**

5 Gene Set Enrichment Analysis (GSEA) **7**

6 Construction of GSEA dotplots **12**

7 Construction of GSEA plots **21**

8 Session info **26**

This file contains the R analysis script used to generate the data and figures for the paper "Identifying SETBP1 haploinsufficiency molecular pathways to improve patient diagnosis using induced pluripotent stem cells and neural disease modelling." The script describes the steps performed after Kallisto transcroipt quantification.

The following sections were each run interactively.

1 Preparation

Beginning with preparing the quantification files.

Loading libraries.

```
library(pacman)

p_load(ggrepel,
       tximport,
       readr,
       RColorBrewer,
       tidyverse,
       GGally,
       limma,
       edgeR,
```

```
        org.Hs.eg.db,
        viridis,
        ComplexUpset,
        DOSE,
        enrichplot,
        clusterProfiler,
        patchwork)
```

Reading in paths to Kallisto files and sample metadata

```
x <- read_csv("./sample_abundance_files.h5", col_names = FALSE) %>%
                                              #This csv contains paths to the abundance files
  rename(path = 1) %>%
  mutate(Sequencing_ID = (path %>% str_remove_all("Kallisto/|/abundance.h5")))

meta_SETBP1_raw <- read_csv("./sample_metadata/SETBP1_RNAseq_samples.csv") # metadata

meta_SETBP1 <- meta_SETBP1_raw %>%
  mutate(Sequencing_ID = str_replace_all(`Sample Name`, " |_|[.]", "-")) %>%
  dplyr::rename(cell_type = `Cell type`) %>%
  select(!`Sample Name`)
```

Preparing DGE object.

```
tx2gene <- read.csv("tx2gene_entrez.csv", header = FALSE)

kallisto_all_samples <- tximport(meta_combined_path$path,
                                 type = "kallisto",
                                 tx2gene = tx2gene,
                                 countsFromAbundance = "lengthScaledTPM",
                                 ignoreAfterBar = TRUE)

DGE <- DGEList(kallisto_all_samples$counts)
DGE$samples <- bind_cols(DGE$samples, meta_SETBP1)
DGE$genes <- bitr(geneID = rownames(DGE),
                  fromType = "ENTREZID",
                  toType = "SYMBOL",
                  OrgDb = org.Hs.eg.db,
                  drop = FALSE)

colnames(DGE$genes) <- c("GeneID", "Symbol")
```

2 Processing DGE object

The DGE object contains the counts data that we will use for analysis. We need to perform processing (filtering/normalization) first.

Filtering of counts data

```
keep.exprs <- filterByExpr(DGE, group = DGE$samples$merged_group)
DGE_filtered <- DGE[keep.exprs, keep.lib.sizes = FALSE]
```

Visualise the results:

```
L <- mean(DGE$samples$lib.size) * 1e-6
M <- median(DGE$samples$lib.size) * 1e-6

cpm <- cpm(DGE)
lcpm <- cpm(DGE, log = T)

lcpm.cutoff <- log2(10/M + 2/L)
nsamples <- ncol(DGE)

col <- inferno(n = nsamples, direction = -1)

par(mfrow=c(1,2))

plot(density(lcpm[,1]),
     col=col[1],
     lwd=2,
     ylim=c(0,0.26),
     las=2,
     main="",
     xlab="")
title(main="A. Raw data", xlab="Log-cpm")

abline(v=lcpm.cutoff, lty=3)
for (i in 2:nsamples){
  den <- density(lcpm[,i])
  lines(den$x, den$y, col=col[i], lwd=2)
}
lcpm <- cpm(DGE_filtered,
            log=TRUE)
plot(density(lcpm[,1]),
     col=col[1],
     lwd=2,
     ylim=c(0,0.26),
     las=2,
     main="",
     xlab="")
title(main="B. Filtered data", xlab="Log-cpm")
abline(v=lcpm.cutoff, lty=3)
for (i in 2:nsamples){
  den <- density(lcpm[,i])
  lines(den$x, den$y, col=col[i], lwd=2)
}
```

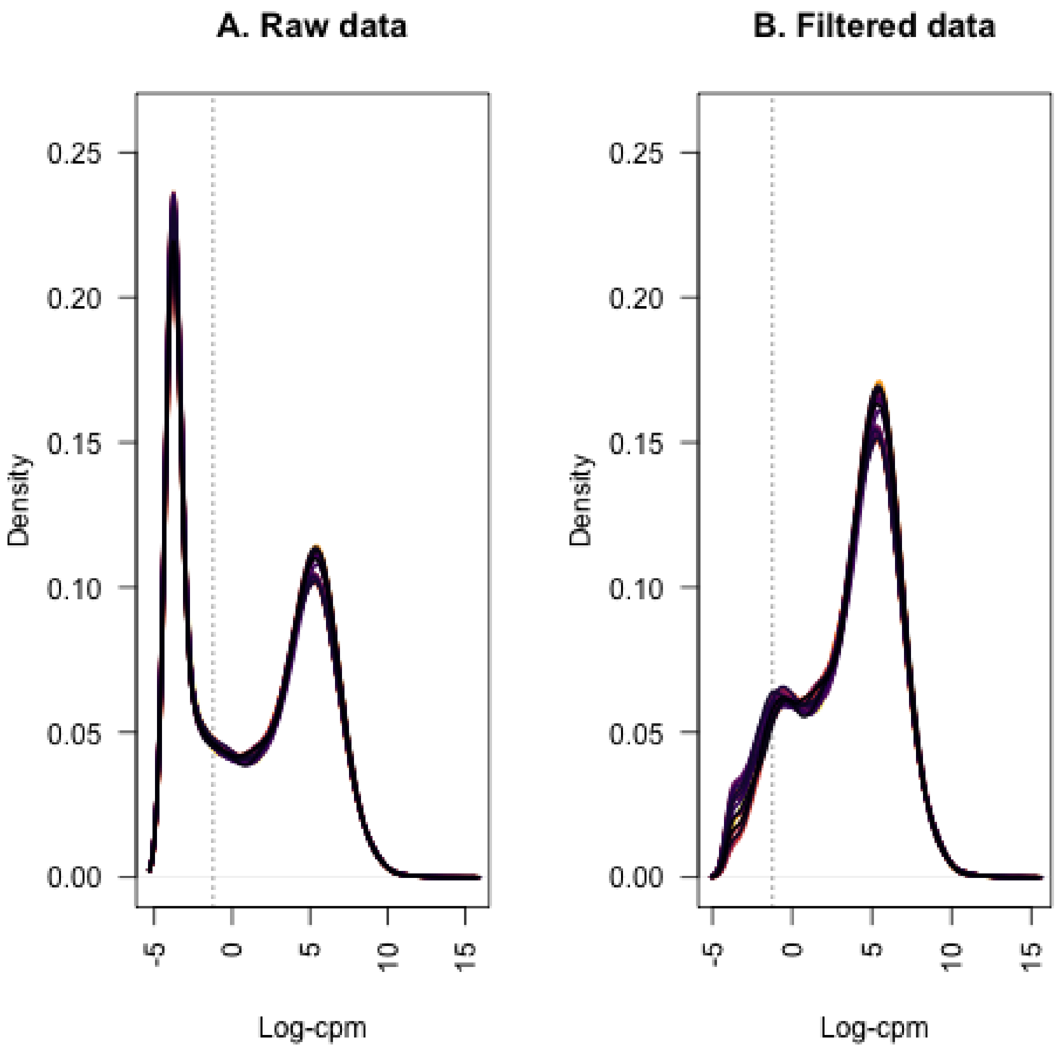


```
DGE_filtered_normalised <- calcNormFactors(DGE_filtered, method = "TMM")

par(mfrow=c(1,2))
lcpm <- cpm(DGE_filtered, log=TRUE)
boxplot(lcpm, las=2, main="")
title(main="A. Example: Unnormalised data",ylab="Log-cpm")
lcpm <- cpm(DGE_filtered_normalised, log=TRUE)
boxplot(lcpm, las=2, main="")
title(main="B. Example: Normalised data",ylab="Log-cpm")
```

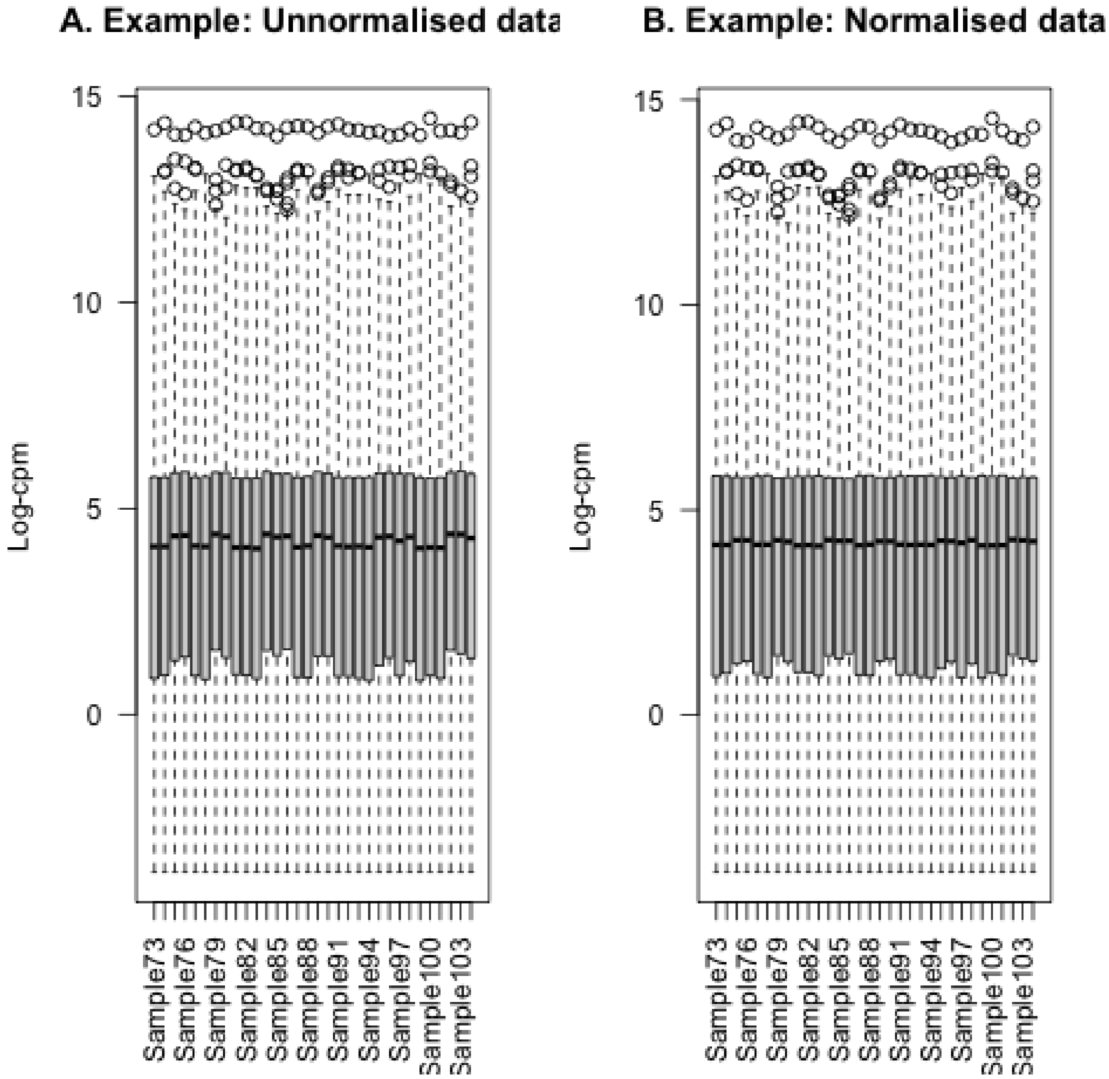

3 Construction of the PCA plot

Following section contains the code required to construct Fig. 3A.

```
lcpm <- cpm(DGE_filtered_normalised, log = T)

col.exp <- DGE_filtered_normalised$samples$cell_type

mds <- plotMDS(lcpm)

toplot <- data.frame(Dim1 = mds$x, Dim2 = mds$y,
                     Group = col.exp)

p <- ggplot(toplot, aes(Dim1, Dim2, colour = col.exp)) +
  geom_point(size = 3) +
```

```
  xlab("PC1 (54.3%)") +
  ylab("PC2 (15.4%)") +
  scale_colour_manual(values = c("blue", "red"),
                      name = "Cell type") +
  theme(axis.title = element_text(size = 12),
        legend.title = element_text(size = 16),
        legend.text = element_text(size = 12))

p
```

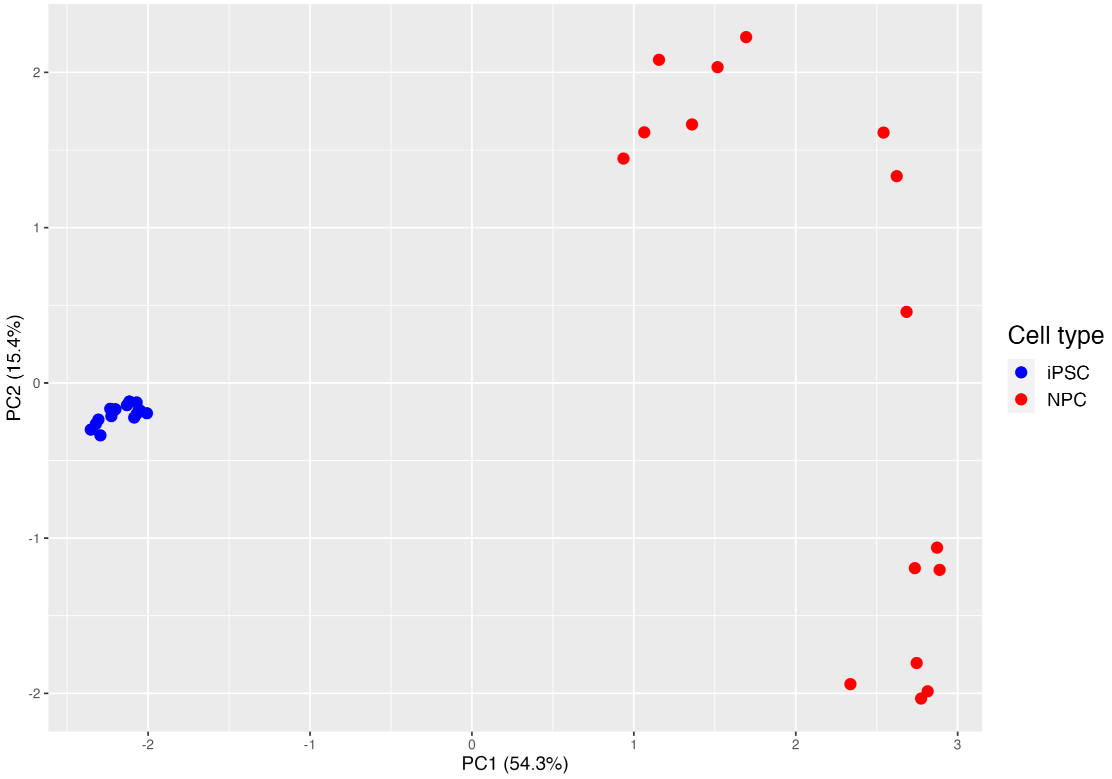

4 Differential gene expression analysis

Perform the DEG analysis. We are interested in the logFC/expression data of the genes in NPCs vs. iPSCs, PATH2 vs. PATH3. vs. VUS2. vs. WT, etc. For example, do we see that markers of neural differentiation are indeed upregulated in the NPCs compared to the iPSCs?

```
# Prepare the experimental design

design <- stats::model.matrix(data = DGE_filtered_normalised$samples, ~0 + merged_group)
colnames(design) <- str_remove_all(colnames(design), "merged_group") # bit of simplifiication

# Construct the voom object
v <- voom(DGE.SETBP1.filtered.normalised, design, plot=TRUE)

# Prepare the contrast matrix. Difference of differences was not used in the final paper.
```

```r
contrast.matrix <- makeContrasts(
  # differentiation
  WT_differentiation = "NPC_WT - iPSC_WT",
  PATH2_differentiation = "NPC_path2 - iPSC_path2",
  PATH3_differentiation = "NPC_path3 - iPSC_path3",
  VUS2_differentiation = "NPC_vus2 - iPSC_vus2",
  # difference between NPCs
  VUS2_WT_NPCcomparison = "NPC_vus2 - NPC_WT",
  PATH2_WT_NPCcomparison = "NPC_path2 - NPC_WT",
  PATH3_WT_NPCcomparison = "NPC_path3 - NPC_WT",
  # difference of differences
  VUS2_DifferenceofDifferences = "(NPC_vus2 - iPSC_vus2) - (NPC_WT - iPSC_WT)",
  PATH2_DifferenceofDifferences = "(NPC_path2 - iPSC_path2) - (NPC_WT - iPSC_WT)",
  PATH3_DifferenceofDifferences = "(NPC_path3 - iPSC_path3) - (NPC_WT - iPSC_WT)",

  levels = colnames(design))

# Perform the linear modelling

vfit <- lmFit(v, design)
vfit <- contrasts.fit(vfit, contrasts = contrast.matrix)

fit <- treat(vfit, lfc = 0.5)
```

Generate logFC lists for use in R. The output can also be saved/exported.

```r
contrasts <- colnames(contrast.matrix)

generateFCdata <- function(x){

SETBP1.logFC.data <- list()

  top <- topTreat(fit, coef = x, number = Inf) %>%
    arrange(desc(logFC)) %>%
    list

  SETBP1.data <- append(SETBP1.logFC.data, top)
}

SETBP1.logFC.data <- sapply(contrasts, generateFCdata)
```

5 Gene Set Enrichment Analysis (GSEA)

Start by preparing input lists for GSEA. This invovles generating the ranked list of genes based on their logFC. In GSEA, we are interested in seeing if particular gene lists (e.g. genes associated with neurodevelopment, neurons, etc.) tend to be represented at the bottom/top of a list. Here, we

are interested in seeing if we can capture enrichments of terms that might be relevant to SETBP1 haploinsufficiency disorder to better understand the biological effects of the p.Thr1387Met (VUS2) variant.

```
GSEA.rank <- function(x){

  genelists <- list()

  top <- topTreat(fit = fit, coef = x, number = Inf) %>%
    arrange(desc(logFC))

  gc <- top$logFC
  names(gc) <- top$GeneID # changed

  genelist <- append(genelists, list(gc))

}

GSEA.lists <- sapply(contrasts, GSEA.rank)
```

Perform GSEA using all GO ontologies, DisGeNET (DGN), and Disease Ontology (DO).

```
SETBP1.DGN.unfiltered <- compareCluster(geneClusters = GSEA.lists,
                     fun = "gseDGN",
                     pvalueCutoff = 1,
                     pAdjustMethod = "BH",
                     nPermSimple = 10000,
                     seed = 42) %>%
  setReadable("org.Hs.eg.db",
              keyType = "ENTREZID")

SETBP1.DO.unfiltered <- compareCluster(fun = "gseDO",
                                   geneClusters = GSEA.lists,
                                   pvalueCutoff = 1,
                                   pAdjustMethod = "BH",
                                   eps = 0,
                                   nPermSimple = 10000,
                                   seed = 42) %>%
  setReadable(OrgDb = org.Hs.eg.db,
              keyType = "ENTREZID")

SETBP1.GO.BP.unfiltered <- compareCluster(fun = "gseGO",
                                   geneClusters = GSEA.lists,
                                   pvalueCutoff = 1,
                                   pAdjustMethod = "BH",
                                   OrgDb = "org.Hs.eg.db",
                                   seed = 42,
                                   eps = 0,
```

```
                                    nPermSimple = 10000,
                                    ont = "BP") %>%
  setReadable(OrgDb = "org.Hs.eg.db")

SETBP1.GO.MF.unfiltered <- compareCluster(fun = "gseGO",
                                    geneClusters = GSEA.lists,
                                    pvalueCutoff = 1,
                                    pAdjustMethod = "BH",
                                    OrgDb = "org.Hs.eg.db",
                                    seed = 42,
                                    eps = 0,
                                    nPermSimple = 10000,
                                    ont = "MF") %>%
  setReadable(OrgDb = "org.Hs.eg.db")

SETBP1.GO.CC.unfiltered <- compareCluster(fun = "gseGO",
                                    geneClusters = GSEA.lists,
                                    pvalueCutoff = 1,
                                    pAdjustMethod = "BH",
                                    OrgDb = "org.Hs.eg.db",
                                    seed = 42,
                                    eps = 0,
                                    nPermSimple = 10000,
                                    ont = "CC") %>%
  setReadable(OrgDb = "org.Hs.eg.db")
```

Save these results.

```
separate.DGN.enrichment <- function(x){
  temp <- SETBP1.DGN.unfiltered@compareClusterResult %>%
    filter(Cluster == x) %>%
    select(!Cluster)

  if(x %in% contrasts.diff){
    write_csv(temp, paste0("./results/results_correctedgenesymbols_23May/SETBP1/gsea_analyses/Di
                           x,
                           "_allDisGeNET.csv"),
              col_names = TRUE)
  }
  if(x %in% contrasts.NPCdiff){
    write_csv(temp, paste0("./results/results_correctedgenesymbols_23May/SETBP1/gsea_analyses/Di
                           x,
                           "_allDisGeNET.csv"),
              col_names = TRUE)
  }
  if(x %in% contrasts.diffofdiff){
    write_csv(temp, paste0("./results/results_correctedgenesymbols_23May/SETBP1/gsea_analyses/Di
                           x,
```

```
                               "_allDisGeNET.csv"),
              col_names = TRUE)
  }
}

map(contrasts, separate.DGN.enrichment)

separate.GO.BP.enrichment <- function(x){
  temp <- SETBP1.GO.BP.unfiltered@compareClusterResult %>%
    filter(Cluster == x) %>%
    select(!Cluster)

  if(x %in% contrasts.diff){
    write_csv(temp,
              paste0("./results/results_correctedgenesymbols_23May/SETBP1/gsea_analyses/GO_terms
  }
  if(x %in% contrasts.NPCdiff){
    write_csv(temp,
              paste0("./results/results_correctedgenesymbols_23May/SETBP1/gsea_analyses/GO_terms
  }
  if(x %in% contrasts.diffofdiff){
    write_csv(temp,
              paste0("./results/results_correctedgenesymbols_23May/SETBP1/gsea_analyses/GO_terms
  }
}

map(contrasts, separate.GO.BP.enrichment)

separate.GO.MF.enrichment <- function(x){
  temp <- SETBP1.GO.MF.unfiltered@compareClusterResult %>%
    filter(Cluster == x) %>%
    select(!Cluster)

  if(x %in% contrasts.diff){
    write_csv(temp,
              paste0("./results/results_correctedgenesymbols_23May/SETBP1/gsea_analyses/GO_terms
  }
  if(x %in% contrasts.NPCdiff){
    write_csv(temp,
              paste0("./results/results_correctedgenesymbols_23May/SETBP1/gsea_analyses/GO_terms
  }
  if(x %in% contrasts.diffofdiff){
    write_csv(temp,
              paste0("./results/results_correctedgenesymbols_23May/SETBP1/gsea_analyses/GO_terms
  }
}

map(contrasts, separate.GO.MF.enrichment)
```

```
separate.GO.CC.enrichment <- function(x){
  temp <- SETBP1.GO.CC.unfiltered@compareClusterResult %>%
    filter(Cluster == x) %>%
    select(!Cluster)

  if(x %in% contrasts.diff){
    write_csv(temp,
              paste0("./results/results_correctedgenesymbols_23May/SETBP1/gsea_analyses/GO_terms
                     x,
                     "_allGO_CC.csv"),
              col_names = TRUE)
  }
  if(x %in% contrasts.NPCdiff){
    write_csv(temp,
              paste0("./results/results_correctedgenesymbols_23May/SETBP1/gsea_analyses/GO_terms
                     x,
                     "_allGO_CC.csv"),
              col_names = TRUE)
  }
  if(x %in% contrasts.diffofdiff){
    write_csv(temp,
              paste0("./results/results_correctedgenesymbols_23May/SETBP1/gsea_analyses/GO_terms
                     x,
                     "_allGO_CC.csv"),
              col_names = TRUE)
  }
}

map(contrasts, separate.GO.CC.enrichment)

separate.DO.enrichment <- function(x){
  temp <- SETBP1.DO.unfiltered@compareClusterResult %>%
    filter(Cluster == x) %>%
    select(!Cluster)

  if(x %in% contrasts.diff){
    write_csv(temp,
              paste0("./results/results_correctedgenesymbols_23May/SETBP1/gsea_analyses/DO_terms
                     x,
                     "_allDO.csv"),
              col_names = TRUE)
  }
  if(x %in% contrasts.NPCdiff){
    write_csv(temp,
              paste0("./results/results_correctedgenesymbols_23May/SETBP1/gsea_analyses/DO_terms
  }
  if(x %in% contrasts.diffofdiff){
```

```
    write_csv(temp,
              paste0("./results/results_correctedgenesymbols_23May/SETBP1/gsea_analyses/DO_terms
                          x,
                          "_allDO.csv"),
              col_names = TRUE)
  }
}

map(contrasts, separate.DO.enrichment)
```

6 Construction of GSEA dotplots

Figures 4 and 5(A-C) contain dotplots depicting results from GSEA. The following section contains the code used to produce these dotplots. The dotplots depict significant enrichments from the GSE, with colour representing the normalised enrichment score, i.e. the direction of the enrichment (do genes tend to be overexpressed or underexpressed?)

For these dotplots, a specified list of terms was provided. These terms are those which are relevant to SETBP1-HD.

Before dotplot construction, some processing was performed to rename the clusters. The resultant dataframes differ from those in the original results in that:

- the Cluster name is simplified
- only difference between NPC results are included (the underlying data is not changed)

This processing is performed below:

```
# Retrieve original results for only difference between NPCs

sapply(c("DisGeNET_terms", "DO_terms"), function(x){

files <- list.files(paste0("./results/results_correctedgenesymbols_23May/SETBP1/gsea_analyses/",
                          x,
                          "/differences_between_NPCs/"),
                    pattern = ".csv",
                    full.names = TRUE)

name <- str_replace_all(x, "_terms", "_files")

assign(x = name,
       value = files,
       envir = .GlobalEnv)
  })

sapply(c("biological_process", "cellular_component", "molecular_function"), function(x){

files <- list.files(paste0("./results/results_correctedgenesymbols_23May/SETBP1/gsea_analyses/GO
                          x,
                          "/differences_between_NPCs/"),
```

```r
                    pattern = ".csv",
                    full.names = TRUE)

name <- paste0(x, "_files")

assign(x = name,
       value = files,
       envir = .GlobalEnv)
  })

names <- c("PATH2",
           "PATH3",
           "VUS2")

# perform renaming

biological_process_df <- data.frame(path = biological_process_files,
                                    name = names)
cellular_component_df <- data.frame(path = cellular_component_files,
                                    name = names)
molecular_function_df <- data.frame(path = molecular_function_files,
                                    name = names)
DisGeNET_function_df <- data.frame(path = DisGeNET_files,
                                   name = names)
DO_df <- data.frame(path = DO_files,
                    name = names)

# Perform the processing

process_csv <- function(path, pattern){

 read_csv(path) %>%
   mutate(Cluster = pattern) %>%
   dplyr::select(Cluster, everything())

    }

dfs <- ls(pattern = "_df$")

DGN_results <- map2_df(DisGeNET_function_df$path, DisGeNET_function_df$name, process_csv)

DO_results <- map2_df(DO_df$path, DO_df$name, process_csv)

GO_MF_results <- map2_df(molecular_function_df$path, molecular_function_df$name, process_csv)

GO_BP_results <- map2_df(biological_process_df$path, biological_process_df$name, process_csv)

GO_CC_results <- map2_df(cellular_component_df$path, cellular_component_df$name, process_csv)
```

```r
# Create a dummy compareCluster object

ck <- compareCluster(geneClusters = GSEA.lists[5:7],
                     fun = "gseDO")
```

The following code section refers to the construction of the dotplot.

```r
# Begin by reading in the list of terms

files <- list.files("./rnaseq_R_code/SETBP1/2024_02_05_FinaliseDotplots/InputLists/",
                    pattern = ".csv",
                    full.names = TRUE)

sapply(files, FUN = function(x){

a <- read_csv(x) %>%
  pull(2)
a <- a[!is.na(a)]

name <- str_replace_all(basename(x), ".csv", "_terms")
assign(value = a,
       x = name,
       envir = .GlobalEnv)

})

# Create the function to construct dotplots

CreateDotplots <- function(results,
                           terms,
                           height = 14,
                           width = 10,
                           margin = c(0.5, 0.5, 0.5, 0.5),
                           name){

temp <- ck
temp@compareClusterResult <- results %>%
  filter(p.adjust < 0.05) %>%
  filter(Description %in% terms)

p <- dotplot(temp,
             showCategory = Inf,
             color = "NES",
             font.size = 14,
             label_format = 100) +
  scale_fill_gradient2(low = "blue",
                       mid = "white",
```

```
                          high = "red") +
  theme(axis.text.x = element_text(angle = 0,
                                    hjust = 0.5,
                                    size = 12),
        axis.text.y = element_text(size = 14),
        plot.margin = unit(margin,
                           "inches")) +
  xlab(NULL)

ggsave(paste0("./rnaseq_R_code/SETBP1/2024_02_05_FinaliseDotplots/Dotplots/", name, ".pdf"),
       p,
       height = height,
       width = width)

ggsave(paste0("./rnaseq_R_code/SETBP1/2024_02_05_FinaliseDotplots/Dotplots/", name, ".png"),
       p,
       height = height,
       width = width)
}


CreateDotplots(results = DGN_results,
               terms = DisGeNET_terms,
               name = "DGN_dotplot",
               margin = c(0.5, 0.25, 0.5, 0.25))

CreateDotplots(results = DO_results,
               terms = DO_terms,
               name = "DO_dotplot",
               margin = c(1.5, 1.9, 1.5, 1.9))

CreateDotplots(results = GO_BP_results,
               terms = GO_BP_terms,
               name = "GO_BP_dotplot",
               margin = c(0, 1.2, 0, 1.2))

CreateDotplots(results = GO_CC_results,
               terms = GO_CC_terms,
               name = "GO_CC_dotplot",
               margin = c(4, 2, 4, 2))

CreateDotplots(results = GO_MF_results,
               terms = GO_MF_terms,
               name = "GO_MF_dotplot",
               margin = c(3.7, 0.5, 3.7, 0.5))
```

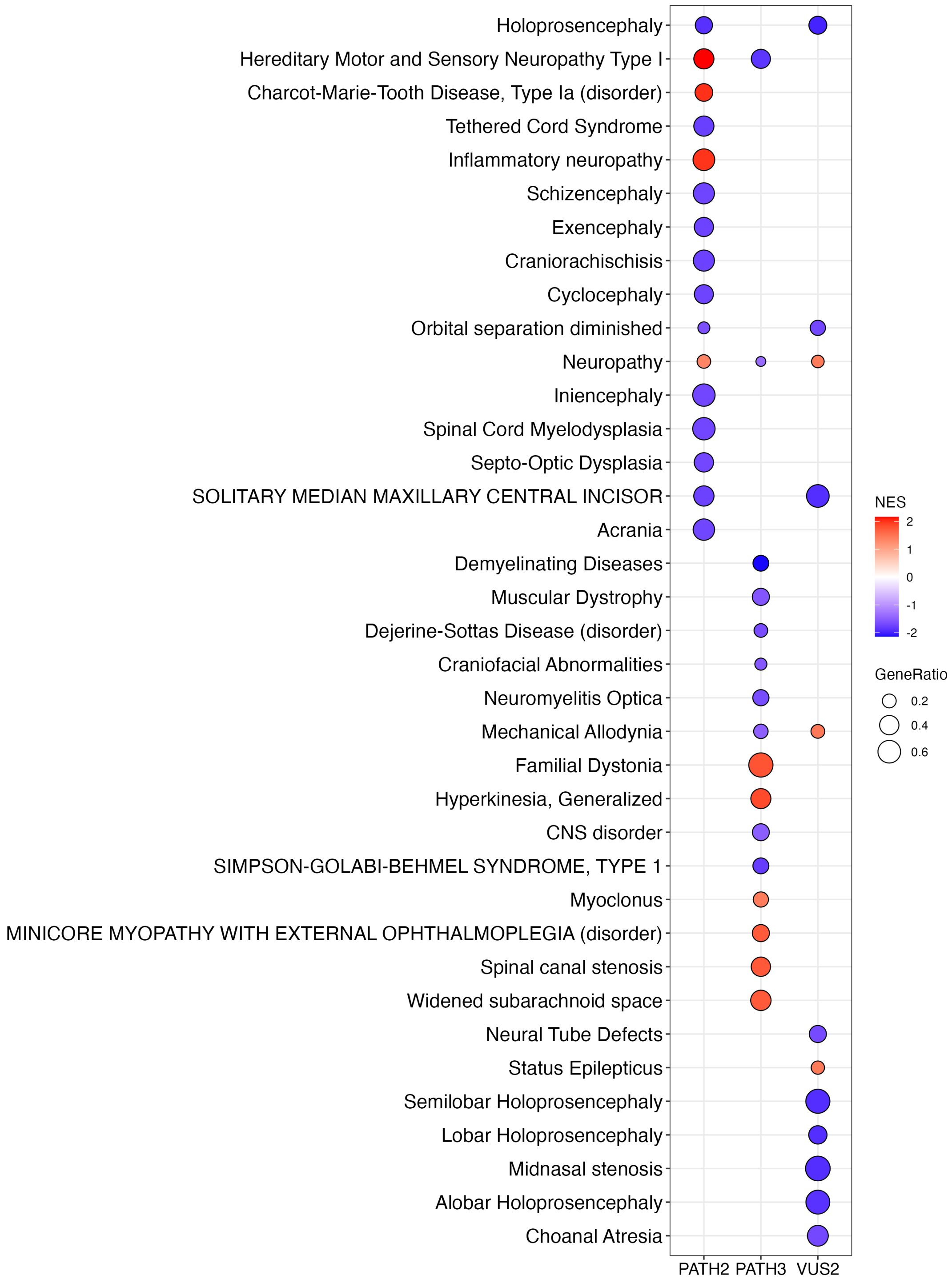
Holoprosencephaly
Hereditary Motor and Sensory Neuropathy Type I
Charcot-Marie-Tooth Disease, Type Ia (disorder)
Tethered Cord Syndrome
Inflammatory neuropathy
Schizencephaly
Exencephaly
Craniorachischisis
Cyclocephaly
Orbital separation diminished
Neuropathy
Iniencephaly
Spinal Cord Myelodysplasia
Septo-Optic Dysplasia
SOLITARY MEDIAN MAXILLARY CENTRAL INCISOR
Acrania
Demyelinating Diseases
Muscular Dystrophy
Dejerine-Sottas Disease (disorder)
Craniofacial Abnormalities
Neuromyelitis Optica
Mechanical Allodynia
Familial Dystonia
Hyperkinesia, Generalized
CNS disorder
SIMPSON-GOLABI-BEHMEL SYNDROME, TYPE 1
Myoclonus
MINICORE MYOPATHY WITH EXTERNAL OPHTHALMOPLEGIA (disorder)
Spinal canal stenosis
Widened subarachnoid space
Neural Tube Defects
Status Epilepticus
Semilobar Holoprosencephaly
Lobar Holoprosencephaly
Midnasal stenosis
Alobar Holoprosencephaly
Choanal Atresia
PATH2
PATH3
VUS2
NES
2
1
0
-1
-2
GeneRatio
0.2
0.4
0.6

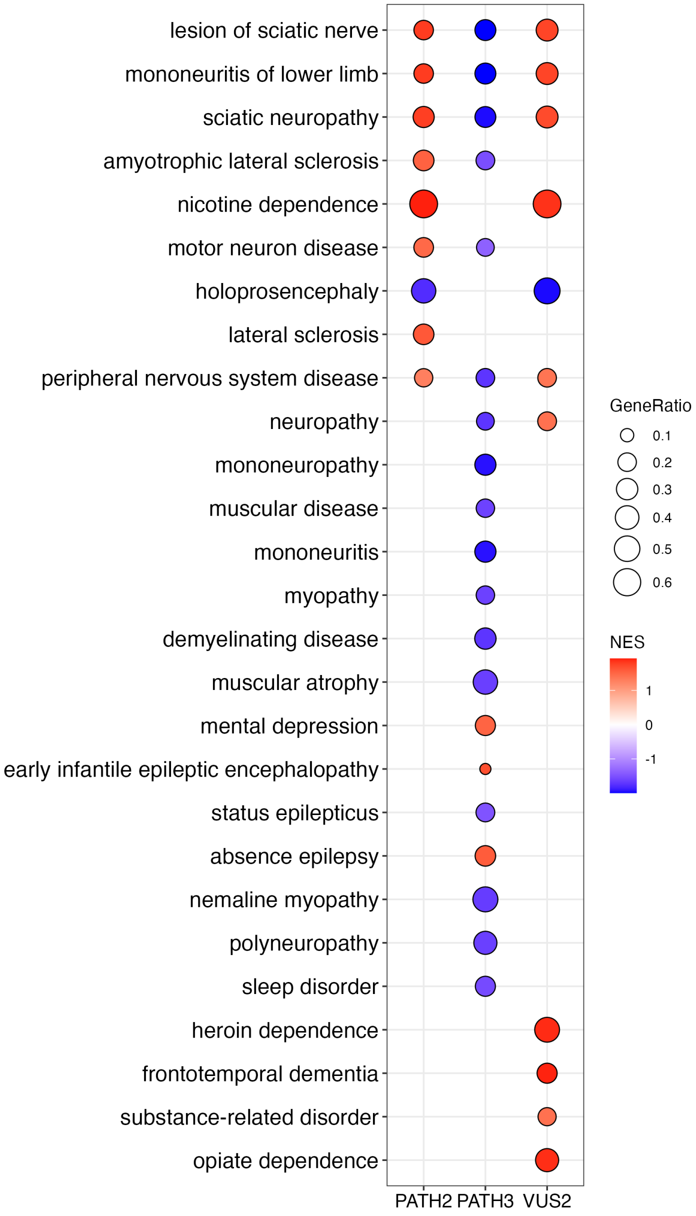
lesion of sciatic nerve
mononeuritis of lower limb
sciatic neuropathy
amyotrophic lateral sclerosis
nicotine dependence
motor neuron disease
holoprosencephaly
lateral sclerosis
peripheral nervous system disease
neuropathy
mononeuropathy
muscular disease
mononeuritis
myopathy
demyelinating disease
muscular atrophy
mental depression
early infantile epileptic encephalopathy
status epilepticus
absence epilepsy
nemaline myopathy
polyneuropathy
sleep disorder
heroin dependence
frontotemporal dementia
substance-related disorder
opiate dependence
PATH2
PATH3
VUS2
GeneRatio
0.1
0.2
0.3
0.4
0.5
0.6
NES
1
0
-1

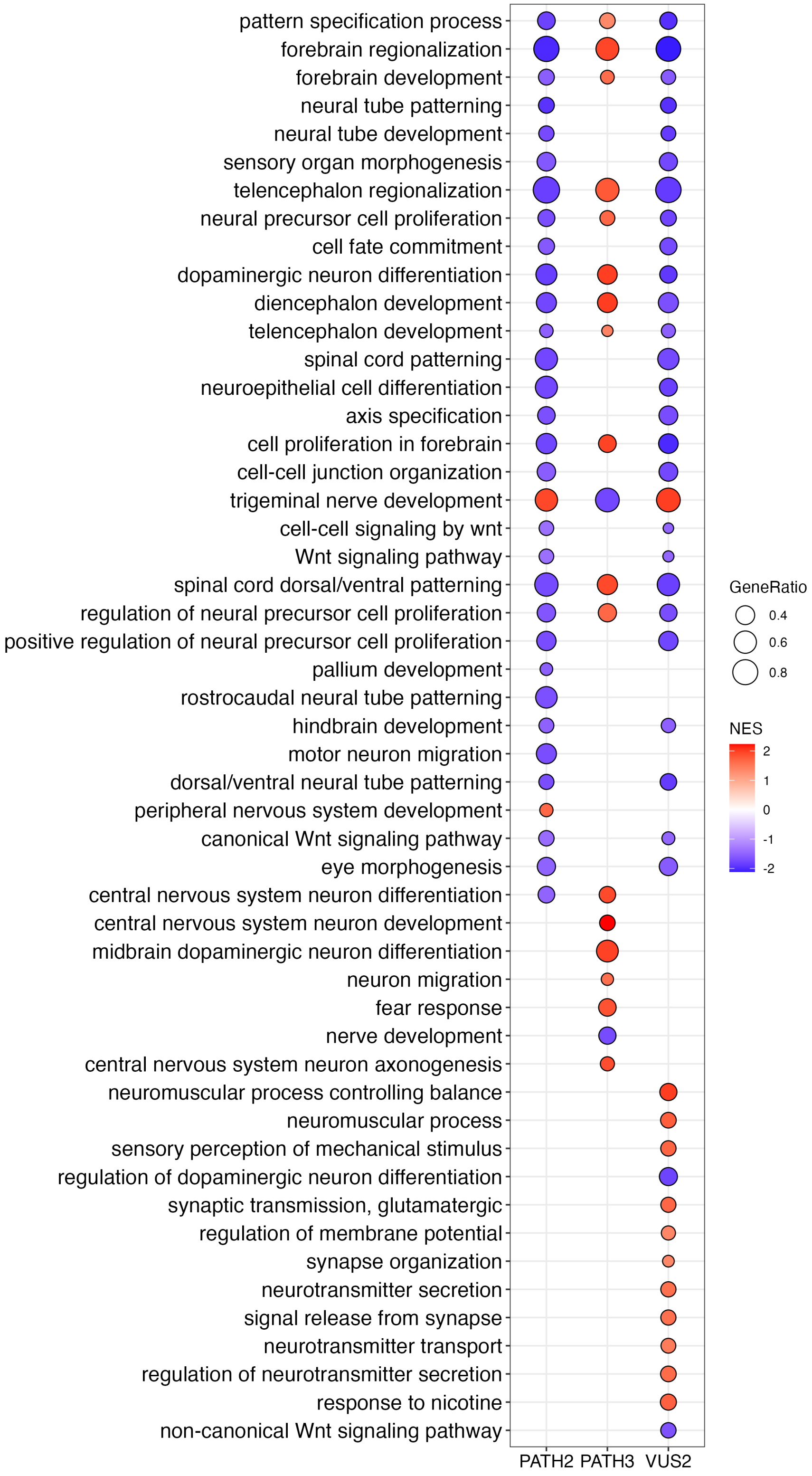
pattern specification process
forebrain regionalization
forebrain development
neural tube patterning
neural tube development
sensory organ morphogenesis
telencephalon regionalization
neural precursor cell proliferation
cell fate commitment
dopaminergic neuron differentiation
diencephalon development
telencephalon development
spinal cord patterning
neuroepithelial cell differentiation
axis specification
cell proliferation in forebrain
cell-cell junction organization
trigeminal nerve development
cell-cell signaling by wnt
Wnt signaling pathway
spinal cord dorsal/ventral patterning
regulation of neural precursor cell proliferation
positive regulation of neural precursor cell proliferation
pallium development
rostrocaudal neural tube patterning
hindbrain development
motor neuron migration
dorsal/ventral neural tube patterning
peripheral nervous system development
canonical Wnt signaling pathway
eye morphogenesis
central nervous system neuron differentiation
central nervous system neuron development
midbrain dopaminergic neuron differentiation
neuron migration
fear response
nerve development
central nervous system neuron axonogenesis
neuromuscular process controlling balance
neuromuscular process
sensory perception of mechanical stimulus
regulation of dopaminergic neuron differentiation
synaptic transmission, glutamatergic
regulation of membrane potential
synapse organization
neurotransmitter secretion
signal release from synapse
neurotransmitter transport
regulation of neurotransmitter secretion
response to nicotine
non-canonical Wnt signaling pathway
PATH2
PATH3
VUS2
GeneRatio
0.4
0.6
0.8
NES
2
1
0
-1
-2

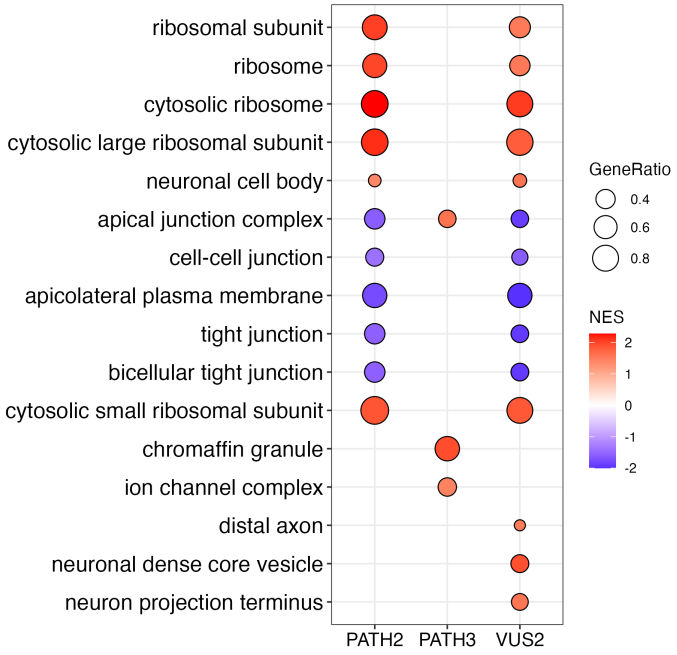
ribosomal subunit
ribosome
cytosolic ribosome
cytosolic large ribosomal subunit
neuronal cell body
apical junction complex
cell-cell junction
apicolateral plasma membrane
tight junction
bicellular tight junction
cytosolic small ribosomal subunit
chromaffin granule
ion channel complex
distal axon
neuronal dense core vesicle
neuron projection terminus
PATH2
PATH3
VUS2
GeneRatio
0.4
0.6
0.8
NES
2
1
0
-1
-2

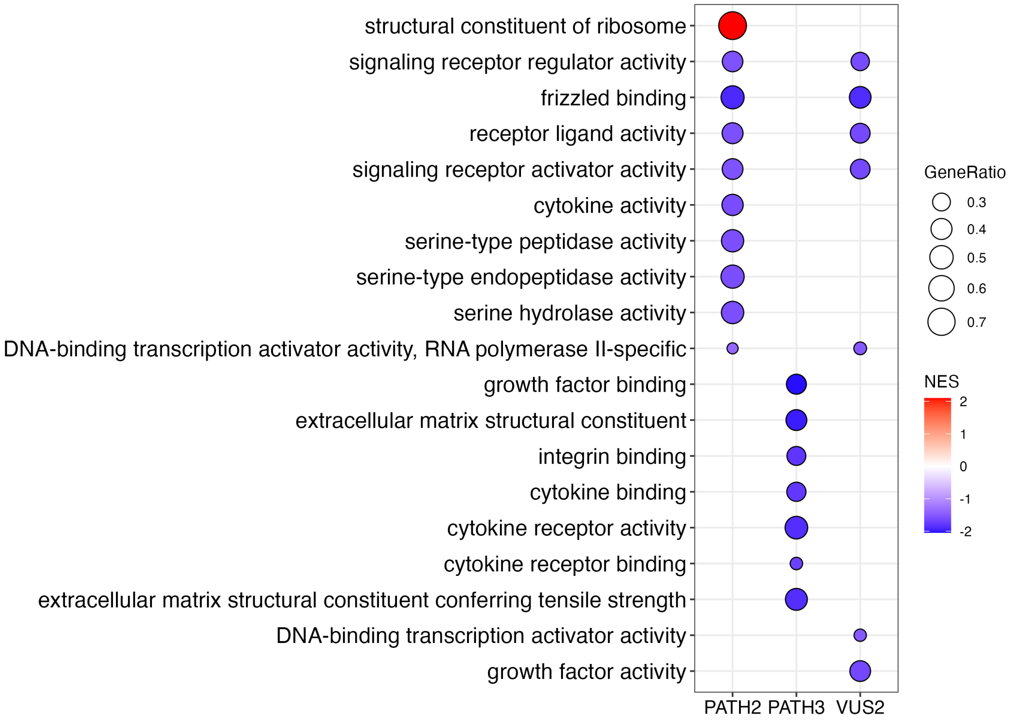
structural constituent of ribosome
signaling receptor regulator activity
frizzled binding
receptor ligand activity
signaling receptor activator activity
cytokine activity
serine-type peptidase activity
serine-type endopeptidase activity
serine hydrolase activity
DNA-binding transcription activator activity, RNA polymerase II-specific
growth factor binding
extracellular matrix structural constituent
integrin binding
cytokine binding
cytokine receptor activity
cytokine receptor binding
extracellular matrix structural constituent conferring tensile strength
DNA-binding transcription activator activity
growth factor activity
PATH2
PATH3
VUS2
GeneRatio
0.3
0.4
0.5
0.6
0.7
NES
2
1
0
-1
-2

7 Construction of GSEA plots

The following section contains code to construct Figure 5(D-F), which are GSEA plots for specified terms. These plots depict the location of the genes in the ranked gene list.

```
# Prepare the list of terms for which a GSEA plot is to be constructed

files <- list.files(path = "./rnaseq_R_code/SETBP1/2023_11_30_GSEAPlots/TermRequests",
                    pattern = ".csv",
                    full.names = TRUE)
files <- files[2:6]

name <- paste0(c("DisGeNET",
          "DO",
          "GO_BP",
          "GO_CC",
          "GO_MF"), "_terms")

data <- data.frame(path = files,
                   name = name)

sapply(1:nrow(data), function(x){

  file <- data[x, 1]
  name <- data[x, 2]

temp <- read_csv(file,
                 col_names = FALSE) %>%
  mutate(ontology = name)
colnames(temp) <- c("ID", "Description", "Ontology")

temp2 <- bind_rows(temp)
str(temp2)

assign(name,
       temp,
       envir = .GlobalEnv)

})

AllGSEA_terms <- bind_rows(GO_BP_terms,
                           GO_MF_terms,
                           GO_CC_terms,
                           DisGeNET_terms,
                           DO_terms)

Path2GO <- gseGO(geneList = GSEA.lists[[6]],
                 ont = "all",
```

```r
                 OrgDb = org.Hs.eg.db,
                 seed = 42)

Path3GO <- gseGO(geneList = GSEA.lists[[7]],
                 ont = "all",
                 OrgDb = org.Hs.eg.db,
                 seed = 42)

VUSGO <- gseGO(geneList = GSEA.lists[[5]],
                 ont = "all",
                 OrgDb = org.Hs.eg.db,
                 seed = 42)

Path2DO <- gseDO(geneList = GSEA.lists[[6]],
                 seed = 42)

Path3DO <- gseDO(geneList = GSEA.lists[[7]],
                 seed = 42)

VUSDO <- gseDO(geneList = GSEA.lists[[5]],
                 seed = 42)

Path2DGN <- gseDGN(geneList = GSEA.lists[[6]],
                 seed = 42)

Path3DGN <- gseDGN(geneList = GSEA.lists[[7]],
                 seed = 42)

VUSDGN <- gseDGN(geneList = GSEA.lists[[5]],
                 seed = 42)
```

The below demonstrates the code used to generate the GSEA plots, of which only three were used for the paper. GSEA

```r
# Example for Molecular Function.

sapply(1:nrow(GO_MF_terms), function(x){


ID <- GO_MF_terms[[x, 1]]
name <- GO_MF_terms[[x, 2]]
name2 <- str_replace_all(GO_MF_terms[[x, 2]], "[ /]", "_")

p1 <- gseaplot(x = Path2GO,
               geneSetID = ID,
               by = "runningScore",
               title = paste0("PATH2 - ", name)) +
  xlab(NULL) +
```

```r
  ylab(NULL)

p2 <- gseaplot(x = Path3GO,
               geneSetID = ID,
               by = "runningScore",
                title = paste0("PATH3 - ", name)) +
  xlab(NULL) +
  theme(axis.title.y = element_text(size = 13))

p3 <- gseaplot(x = VUSGO,
               geneSetID = ID,
               by = "runningScore",
                title = paste0("VUS2 - ", name)) +
  ylab(NULL)

final_p <- wrap_plots(p1, p2, p3,
                      ncol = 1)

ggsave(paste0("./rnaseq_R_code/SETBP1/2023_11_30_GSEAPlots/GSEAPlots/GO_MF/", name2, "_GSEAPlot.
       final_p)
})
```

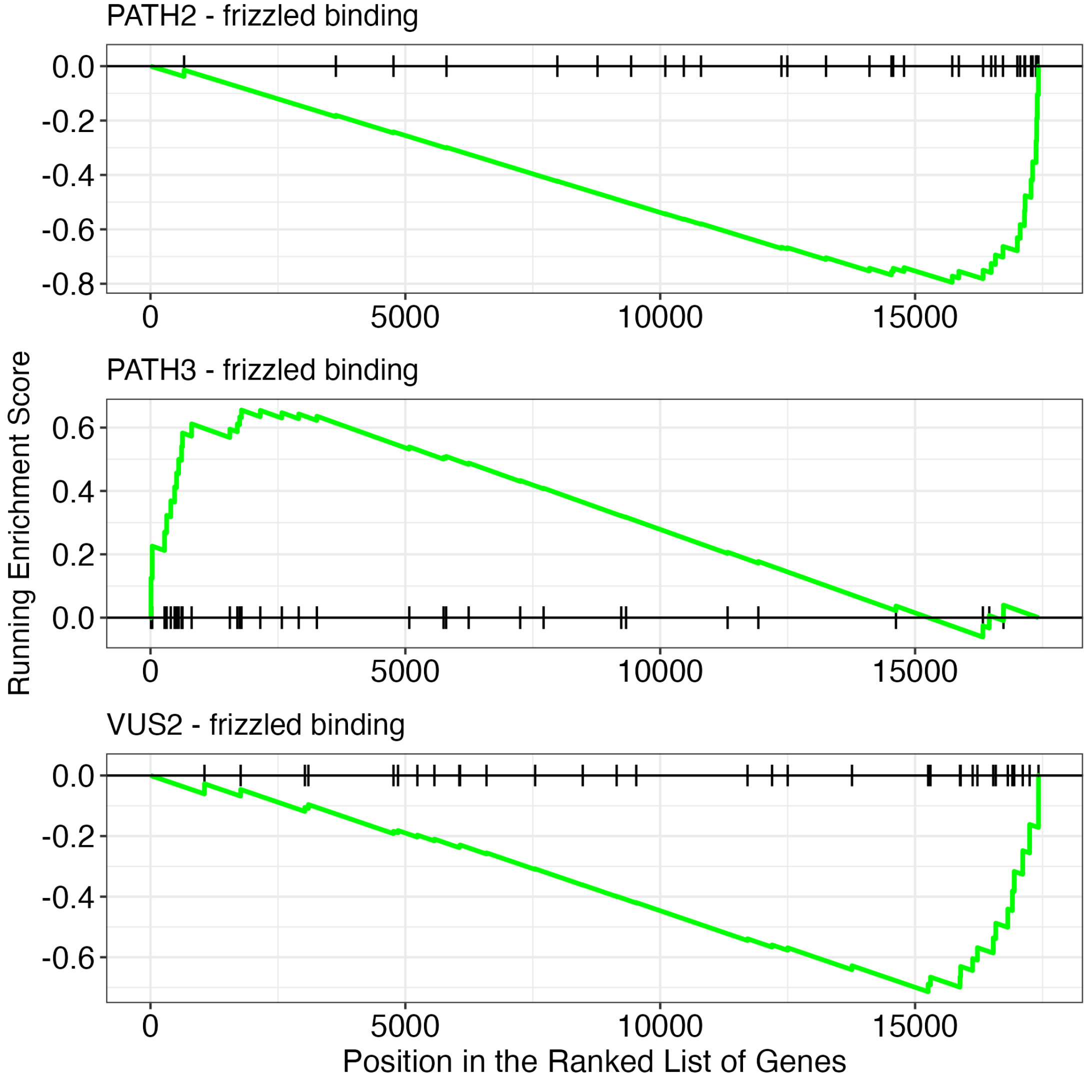
PATH2 - frizzled binding
PATH3 - frizzled binding
VUS2 - frizzled binding
Running Enrichment Score
Position in the Ranked List of Genes
0.0
-0.2
-0.4
-0.6
-0.8
0.6
0.4
0.2
0
5000
10000
15000

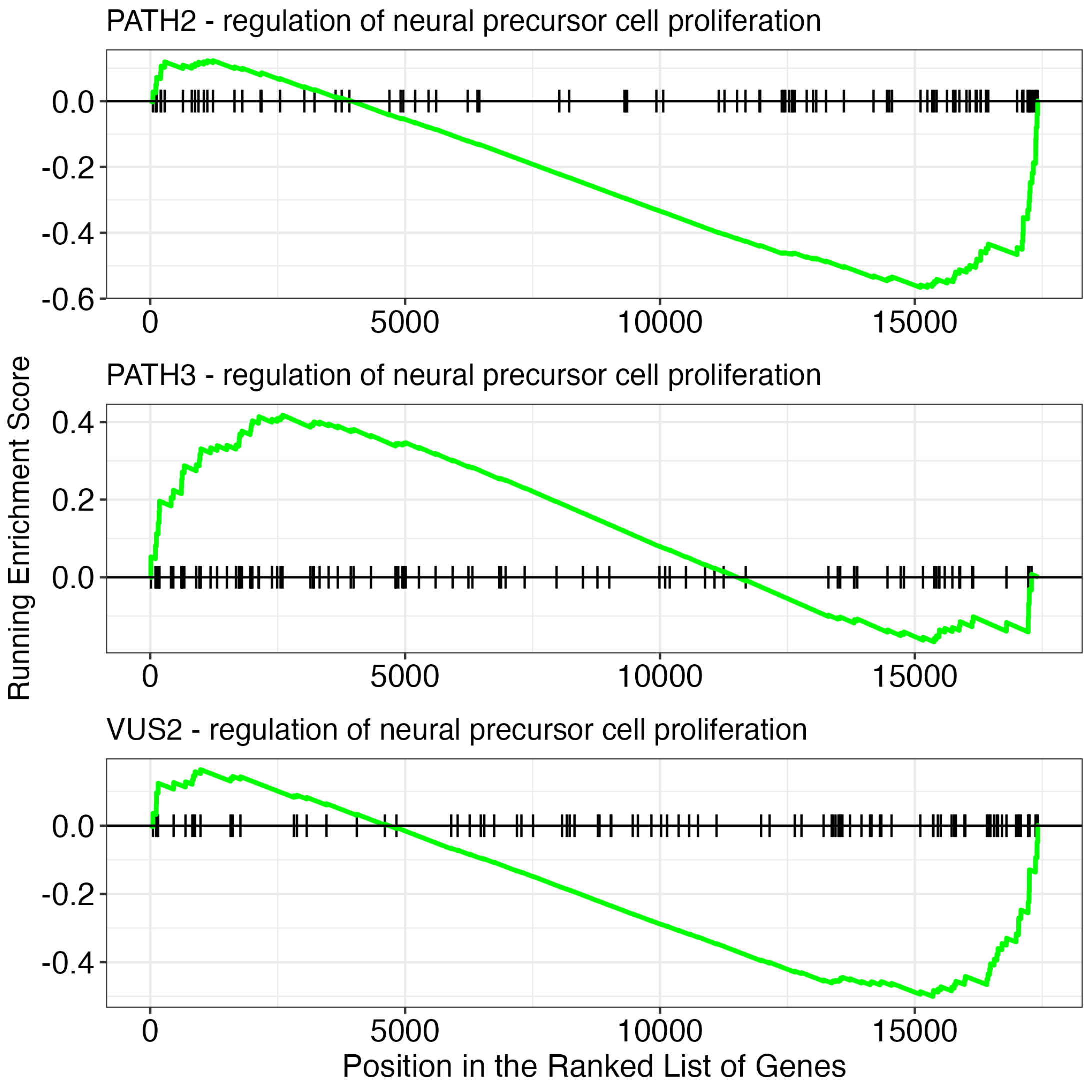
PATH2 - regulation of neural precursor cell proliferation
0.0
-0.2
-0.4
-0.6
0
5000
10000
15000
PATH3 - regulation of neural precursor cell proliferation
0.4
0.2
0.0
0
5000
10000
15000
VUS2 - regulation of neural precursor cell proliferation
0.0
-0.2
-0.4
0
5000
10000
15000
Running Enrichment Score
Position in the Ranked List of Genes

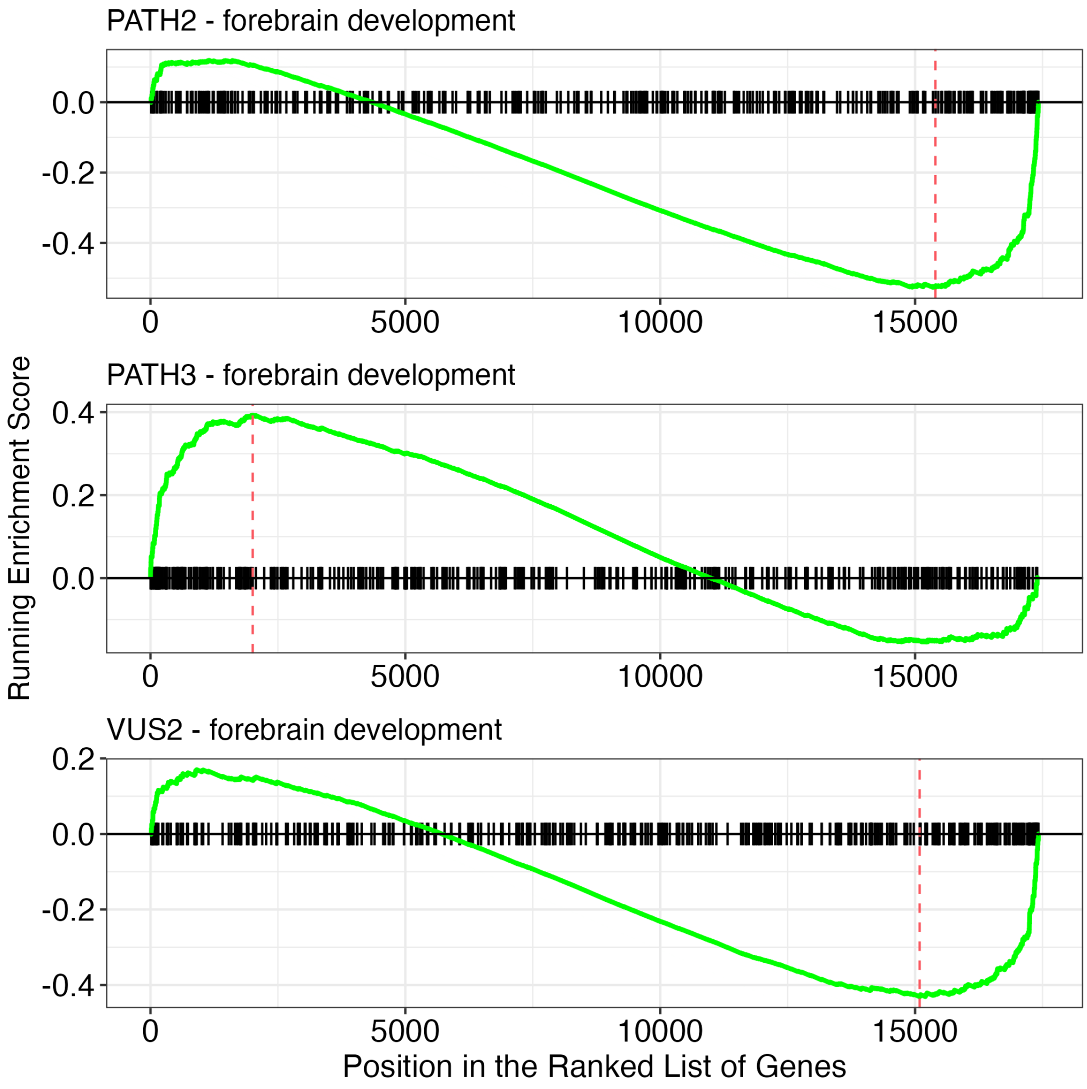

8 Session info

```
sessionInfo()
```

```
R version 4.3.2 (2023-10-31)
Platform: aarch64-apple-darwin23.0.0 (64-bit)
Running under: macOS Sonoma 14.0

Matrix products: default
BLAS:   /opt/homebrew/Cellar/openblas/0.3.25/lib/libopenblasp-r0.3.25.dylib
LAPACK: /opt/homebrew/Cellar/r/4.3.2/lib/R/lib/libRlapack.dylib;  LAPACK version 3.11.0

locale:
[1] en_AU.UTF-8/en_AU.UTF-8/en_AU.UTF-8/C/en_AU.UTF-8/en_AU.UTF-8
```

```
time zone: Australia/Perth
tzcode source: internal

attached base packages:
[1] stats4    stats     graphics  grDevices utils     datasets  methods
[8] base

other attached packages:
 [1] patchwork_1.1.3          clusterProfiler_4.8.2
 [3] enrichplot_1.20.3        DOSE_3.26.1
 [5] ComplexUpset_1.3.3       viridis_0.6.4
 [7] viridisLite_0.4.2        org.Hs.eg.db_3.18.0
 [9] AnnotationDbi_1.64.1     IRanges_2.36.0
[11] S4Vectors_0.40.2         Biobase_2.62.0
[13] BiocGenerics_0.48.1      edgeR_3.42.4
[15] limma_3.58.1             GGally_2.2.0
[17] lubridate_1.9.3          forcats_1.0.0
[19] stringr_1.5.1            dplyr_1.1.4
[21] purrr_1.0.2              tidyr_1.3.0
[23] tibble_3.2.1             tidyverse_2.0.0
[25] RColorBrewer_1.1-3       readr_2.1.4
[27] tximport_1.28.0          ggrepel_0.9.4
[29] ggplot2_3.4.4            pacman_0.5.1

loaded via a namespace (and not attached):
 [1] DBI_1.1.3               bitops_1.0-7            shadowtext_0.1.2
 [4] gson_0.1.0              gridExtra_2.3           rlang_1.1.3
 [7] magrittr_2.0.3          compiler_4.3.2          RSQLite_2.3.4
[10] png_0.1-8               vctrs_0.6.5             reshape2_1.4.4
[13] pkgconfig_2.0.3         crayon_1.5.2            fastmap_1.1.1
[16] XVector_0.42.0          ggraph_2.1.0            utf8_1.2.4
[19] HDO.db_0.99.1           tzdb_0.4.0              bit_4.0.5
[22] zlibbioc_1.48.0         cachem_1.0.8            aplot_0.2.2
[25] GenomeInfoDb_1.38.1     jsonlite_1.8.8          blob_1.2.4
[28] BiocParallel_1.36.0     tweenr_2.0.2            parallel_4.3.2
[31] R6_2.5.1                stringi_1.8.3           GOSemSim_2.28.0
[34] Rcpp_1.0.12             timechange_0.2.0        Matrix_1.6-3
[37] splines_4.3.2           igraph_1.5.1            tidyselect_1.2.0
[40] qvalue_2.32.0           codetools_0.2-19        lattice_0.21-9
[43] plyr_1.8.9              treeio_1.26.0           withr_2.5.2
[46] KEGGREST_1.42.0         gridGraphics_0.5-1      ggstats_0.5.1
[49] scatterpie_0.2.1        polyclip_1.10-6         Biostrings_2.70.1
[52] pillar_1.9.0            ggtree_3.10.0           ggfun_0.1.3
[55] generics_0.1.3          RCurl_1.98-1.13         hms_1.1.3
[58] munsell_0.5.0           scales_1.3.0            tidytree_0.4.5
[61] glue_1.7.0              lazyeval_0.2.2          tools_4.3.2
[64] data.table_1.14.10      locfit_1.5-9.8          fgsea_1.26.0
[67] fs_1.6.3                graphlayouts_1.0.2      fastmatch_1.1-4
```

```
[70] tidygraph_1.2.3         cowplot_1.1.1           grid_4.3.2
[73] ape_5.7-1               colorspace_2.1-0        nlme_3.1-163
[76] GenomeInfoDbData_1.2.11 ggforce_0.4.1           cli_3.6.2
[79] fansi_1.0.6             gtable_0.3.4            yulab.utils_0.1.1
[82] digest_0.6.34           ggplotify_0.1.2         farver_2.1.1
[85] memoise_2.0.1           lifecycle_1.0.4         httr_1.4.7
[88] statmod_1.5.0           GO.db_3.17.0            bit64_4.0.5
[91] MASS_7.3-60
```
