# Supplementary material for: Identifying SETBP1 haploinsufficiency molecular pathways to improve patient diagnosis using induced pluripotent stem cells and neural disease modelling
Source: Mol Autism. 2024 Sep 30;15:42. doi: 10.1186/s13229-024-00625-1 (PMC11443744; doi:10.1186/s13229-024-00625-1)
Supplement: Supplementary file 3 — Additional file 3 [file 13229_2024_625_MOESM3_ESM.pdf]

A

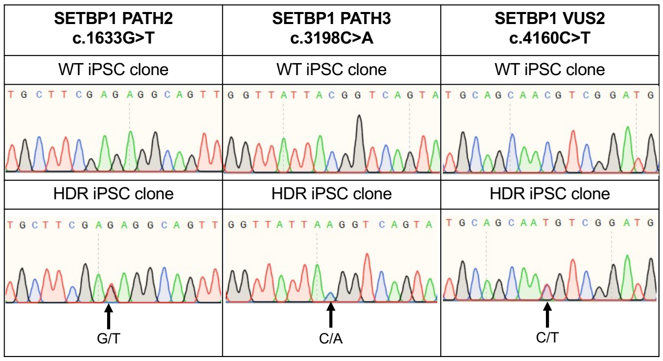

B

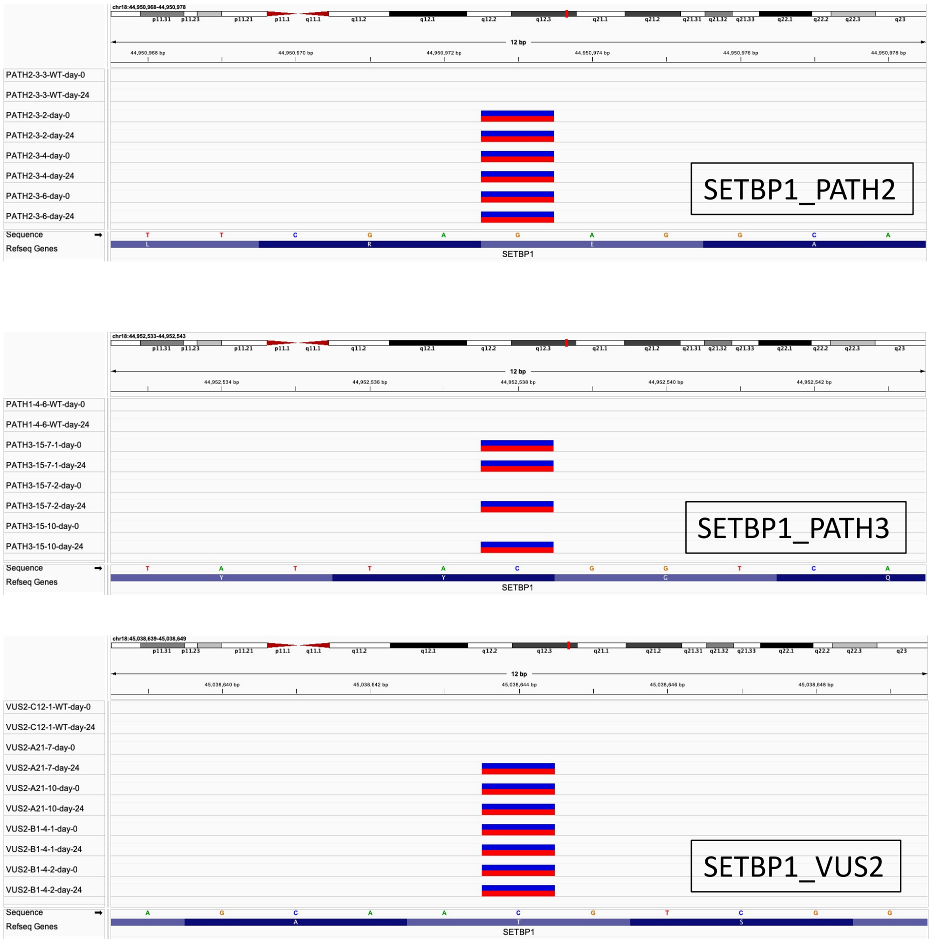

C

Clone	SETBP1 variant	Genotype	Reference	Alternate	Quality
PATH2-1	c.1633G>T	HDR/WT	G	T	316.64
PATH2-2	c.1633G>T	HDR/WT	G	T	184.64
PATH2-3	c.1633G>T	HDR/WT	G	T	121.64
PATH3-1	c.3198C>A	HDR/WT	C	A	94.64
PATH3-2	c.3198C>A	HDR/WT	C	A	
VUS2-1	c.4160C>T	HDR/WT	C	T	
VUS2-2	c.4160C>T	HDR/WT	C	T	105.64
VUS2-3	c.4160C>T	HDR/WT	C	T	146.64

Supplementary Figure 1. SETBP1 genetic variant expression in clonal cell lines. A. Sanger sequencing on gDNA indicates heterozygous SETBP1 genetic variant clones. **B.** SETBP1 - PATH2, PATH3, and VUS2 clones (iPSCs and NPCs) transcripts were aligned to the GRCh38 reference genome for detection of the respective genetic variants. Red and blue boxes indicate mismatches to reference sequence on both the positive and negative strand, indicating the presence of the variant in the sample. **C.** Table of variant calling metrics for SETBP1 in iPSCs samples. Quality: Phred-scaled quality score.

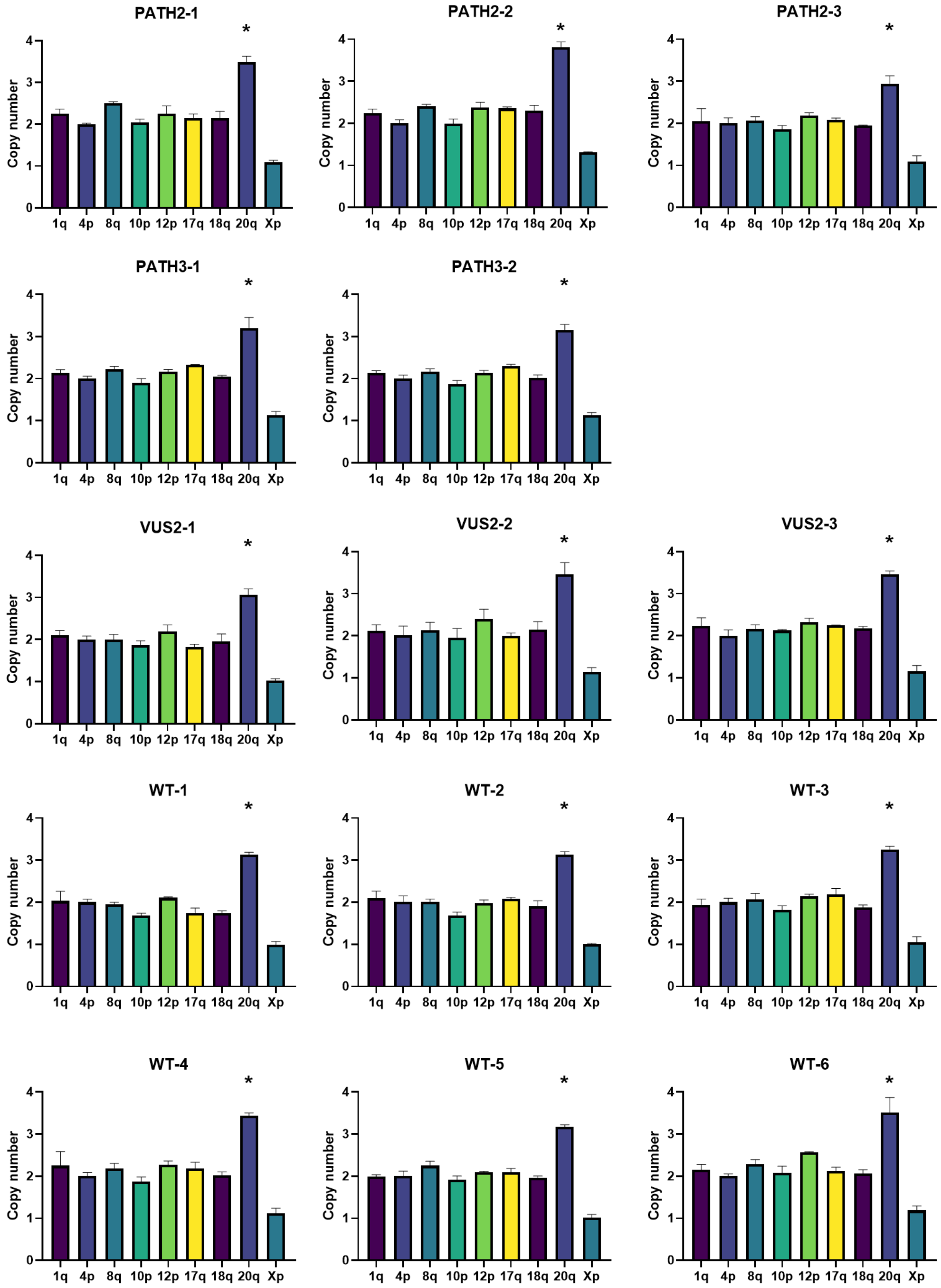

Supplementary Figure 2. Screening of iPSC clones for common chromosomal abnormalities. PATH2, PATH3, VUS2 and WT iPSC clones were screened by qPCR for abnormalities in chromosomal regions prone to genomic instability in iPSCs. Copy numbers were not significantly different from 2 for each chromosomal region in 1q, 4p, 8q, 12p, 17q, 18q or 1 for chromosomal region in Xp. An amplification in chromosomal region 20q was identified in all iPSC clones. p*<0.05

	Off-target site 1	Off-target site 2
	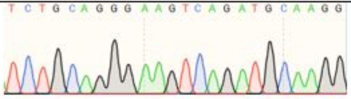	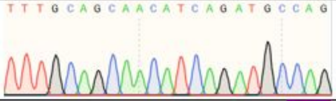
**WT sequence**	TCTGCAGGGAAGTCAGATGCAAG	TTTGCAGCAACATCAGATGCCAG
**VUS2-1**	TCTGCAGGGAAGTCAGATGCAAG	TTTGCAGCAACATCAGATGCCAG
**VUS2-2**	TCTGCAGGGAAGTCAGATGCAAG	TTTGCAGCAACATCAGATGCCAG
**VUS2-3**	TCTGCAGGGAAGTCAGATGCAAG	TTTGCAGCAACATCAGATGCCAG

	Off-target site 3	Off-target site 4
	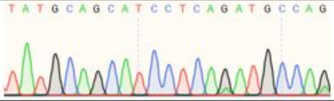	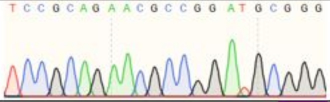
**WT sequence**	TATGCAGCATCCTCAGATGCCAG	TCCGCAGAACGCCGGATGCGGG
**VUS2-1**	TATGCAGCATCCTCAGATGCCAG	TCCGCAGAACGCCGGATGCGGG
**VUS2-2**	TATGCAGCATCCTCAGATGCCAG	TCCGCAGAACGCCGGATGCGGG
**VUS2-3**	TATGCAGCATCCTCAGATGCCAG	TCCGCAGAACGCCGGATGCGGG

	Off-target site 5	Off-target site 6
	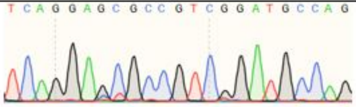	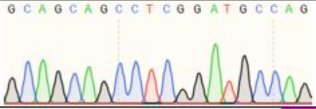
**WT sequence**	TCAGGAGCGCCGTCGGATGCCAG	GCAGCAGCAGCCTCGGATGCCAG
**VUS2-1**	TCAGGAGCGCCGTCGGATGCCAG	GCAGCAGCAGCCTCGGATGCCAG
**VUS2-2**	TCAGGAGCGCCGTCGGATGCCAG	GCAGCAGCAGCCTCGGATGCCAG
**VUS2-3**	TCAGGAGCGCCGTCGGATGCCAG	GCAGCAGCAGCCTCGGATGCCAG

Supplementary Figure 3. Sanger sequencing of CRISPR/Cas9 off-target sites for SETBP1 VUS2 crRNA. Sequences upstream of PAM region (highlighted in pink) remain unmodified in iPSC clones indicating no off-target endonuclease activity of Cas9 enzyme.

	Off-target site 1	Off-target site 2
	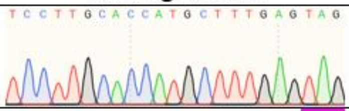	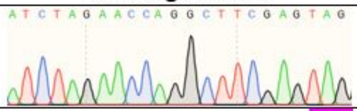
**WT sequence**	TCCTTGCACCATGCTTTGAGTAG	ATCTAGAACCAGGCTTCGAGTAG
**PATH2-1**	TCCTTGCACCATGCTTTGAGTAG	ATCTAGAACCAGGCTTCGAGTAG
**PATH2-2**	TCCTTGCACCATGCTTTGAGTAG	ATCTAGAACCAGGCTTCGAGTAG
**PATH2-3**	TCCTTGCACCATGCTTTGAGTAG	ATCTAGAACCAGGCTTCGAGTAG

	Off-target site 3	Off-target site 4
	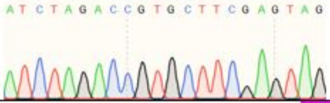	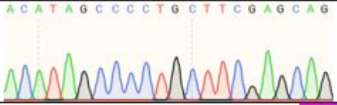
**WT sequence**	ATCTAGACCGTGCTTCGAGTAG	ACATAGCCCCTGCTTCGAGCAG
**PATH2-1**	ATCTAGACCGTGCTTCGAGTAG	ACATAGCCCCTGCTTCGAGCAG
**PATH2-2**	ATCTAGACCGTGCTTCGAGTAG	ACATAGCCCCTGCTTCGAGCAG
**PATH2-3**	ATCTAGACCGTGCTTCGAGTAG	ACATAGCCCCTGCTTCGAGCAG

	Off-target site 5	Off -target site 6
	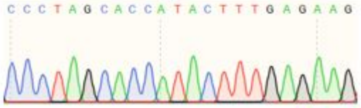	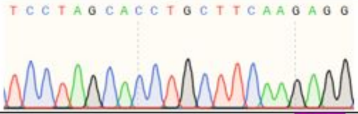
**WT sequence**	CCCTAGCACCATACTTTGAGAAG	TCCTAGCACCTGCTTCAAGAGG
**PATH2-1**	CCCTAGCACCATACTTTGAGAAG	TCCTAGCACCTGCTTCAAGAGG
**PATH2-2**	CCCTAGCACCATACTTTGAGAAG	TCCTAGCACCTGCTTCAAGAGG
**PATH2-3**	CCCTAGCACCATACTTTGAGAAG	TCCTAGCACCTGCTTCAAGAGG

Supplementary Figure 4. Sanger sequencing of CRISPR/Cas9 off-target sites for SETBP1 PATH2 crRNA. Sequences upstream of PAM region (highlighted in pink) remain unmodified in iPSC clones indicating no off-target endonuclease activity of Cas9 enzyme.

	Off-target site 1	Off-target site 2
	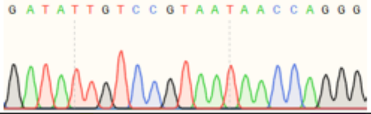	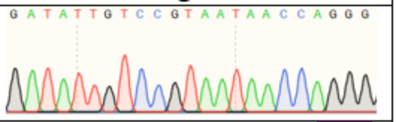
**WT sequence**	GATATTGTCCGTAATAACCAGGG	GATATTGTCCGTAATAACCAGGG
**PATH3-1**	GATATTGTCCGTAATAACCAGGG	GATATTGTCCGTAATAACCAGGG
**PATH3-2**	GATATTGTCCGTAATAACCAGGG	GATATTGTCCGTAATAACCAGGG

	Off-target site 3	Off-target site 4
	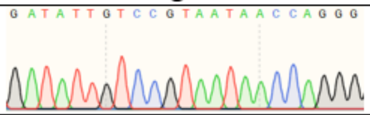	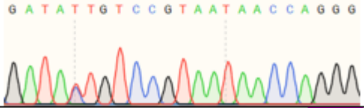
**WT sequence**	GATATTGTCCGTAATAACCAGGG	GATATTGTCCGTAATAACCAGGG
**PATH3-1**	GATATTGTCCGTAATAACCAGGG	GATATTGTCCGTAATAACCAGGG
**PATH3-2**	GATATTGTCCGTAATAACCAGGG	GATATTGTCCGTAATAACCAGGG

	Off-target site 5	Off-target site 6
	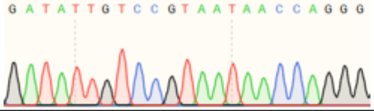	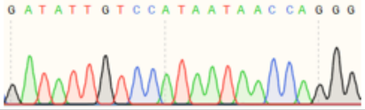
**WT sequence**	GATATTGTCCGTAATAACCAGGG	GATATTGTCCATAATAACCAGGG
**PATH3-1**	GATATTGTCCGTAATAACCAGGG	GATATTGTCCATAATAACCAGGG
**PATH3-2**	GATATTGTCCGTAATAACCAGGG	GATATTGTCCATAATAACCAGGG

Supplementary Figure 5. Sanger sequencing of CRISPR/Cas9 off-target sites for SETBP1 PATH3 crRNA. Sequences upstream of PAM region (highlighted in pink) remain unmodified in iPSC clones indicating no off-target endonuclease activity of Cas9 enzyme.

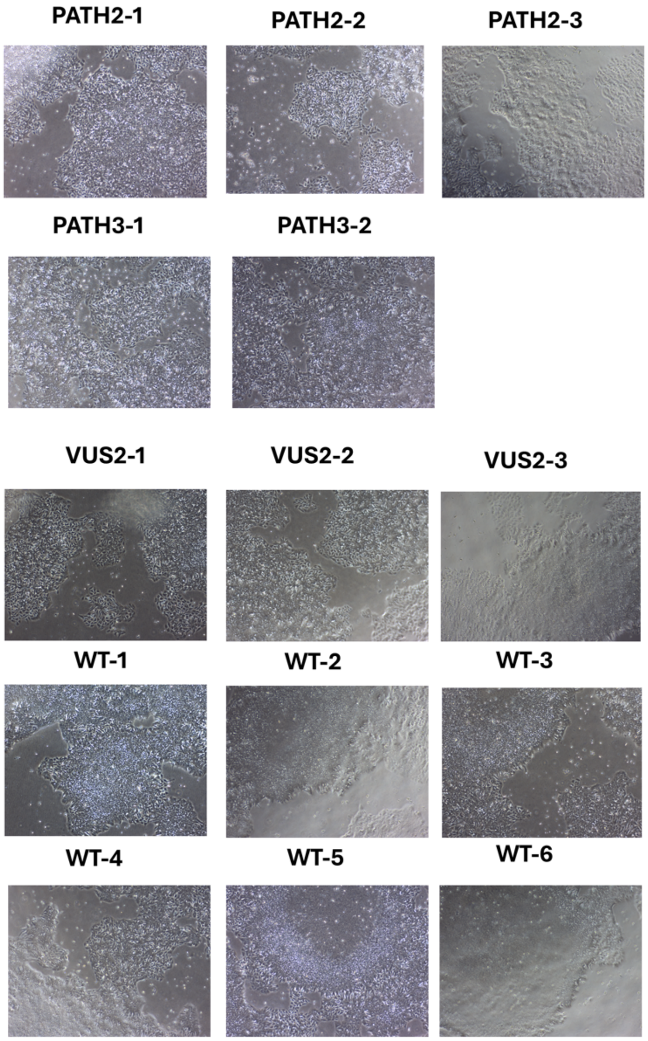

Supplementary Figure 6. Morphology of SETBP1 iPSC clones. SETBP1 PATH2, PATH3 or VUS2 iPSCs did not appear morphologically different to WT clones. Morphology was also indistinguishable from the KOLF2 parental iPSC line. Images captured at 4x objective magnification.

A

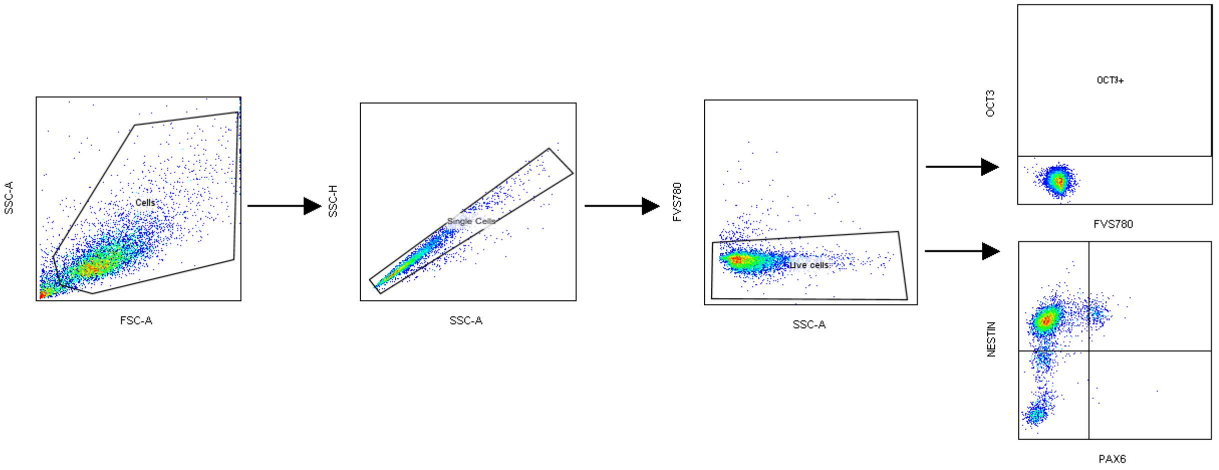

B

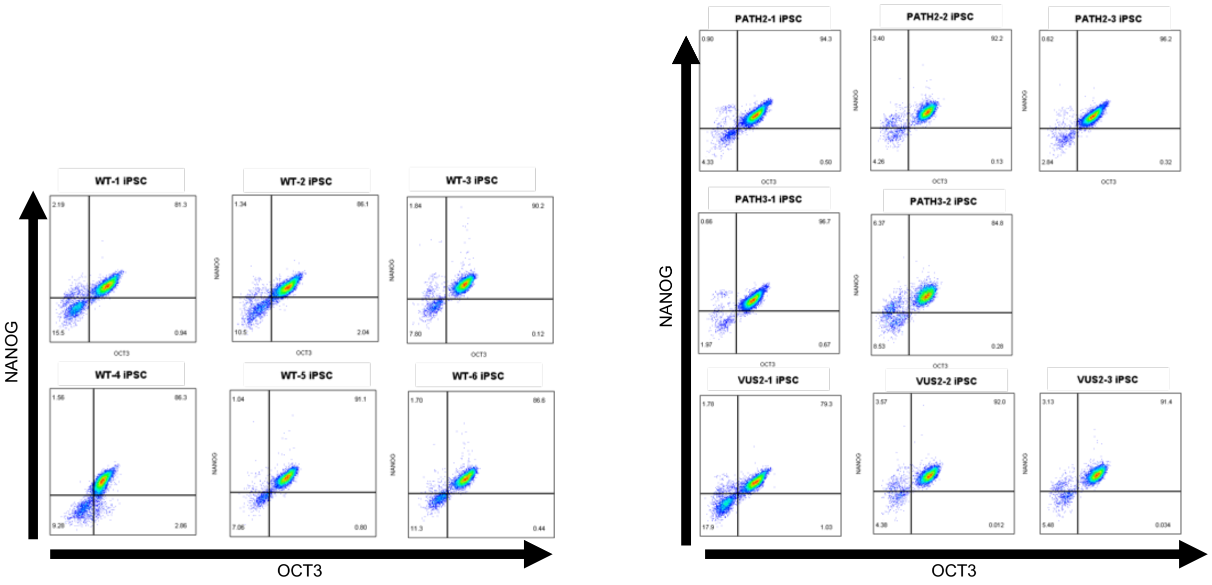

Supplementary Figure 7. Gating strategy for flow cytometry analysis, and stem cell marker expression in clonal cell lines. (A) Cell populations expressing pluripotency and neural markers were analysed after gating to exclude debris (FSC vs. SSC), doublets (SSC-H vs. SSC-A) and dead cells (FVS780 vs. SSC) for iPSCs and neural cells. Pluripotent cells were identified by OCT-3/4 expression and neural progenitor cells identified by Nestin and PAX6 expression. (B) SETBP1 PATH2, PATH3, VUS2 and WT iPSC line was assessed for OCT3 and NANOG co-expression prior to neural differentiation induction. The percentage of live cell co-expressing OCT3 and NANOG ranged from 79.3% - 96.7%.

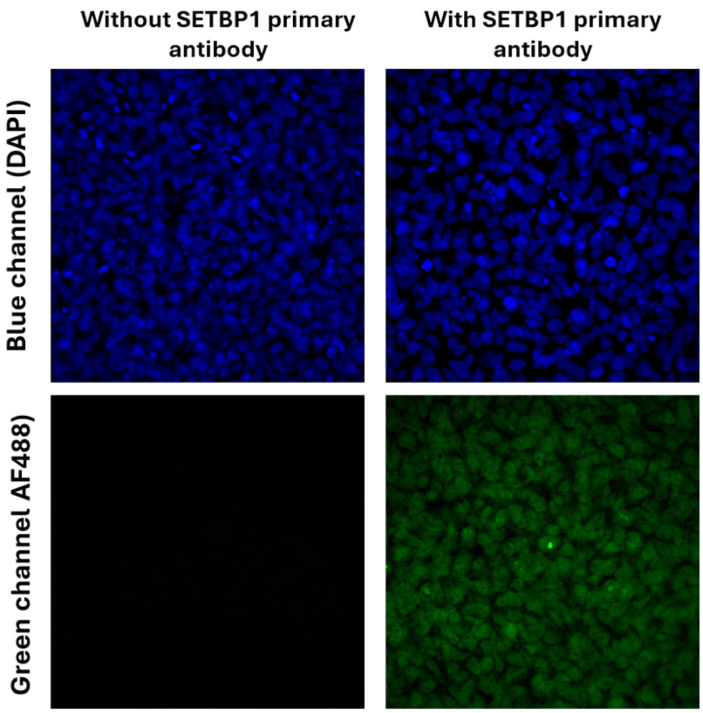

Supplementary Figure 8. Specificity of SETBP1 immunostaining. No signal in the green channel was detected for the negative control conditions. Cells in both antibody conditions are VUS2-3 NPCs. Images were captured using the same microscope settings and images processed identically.

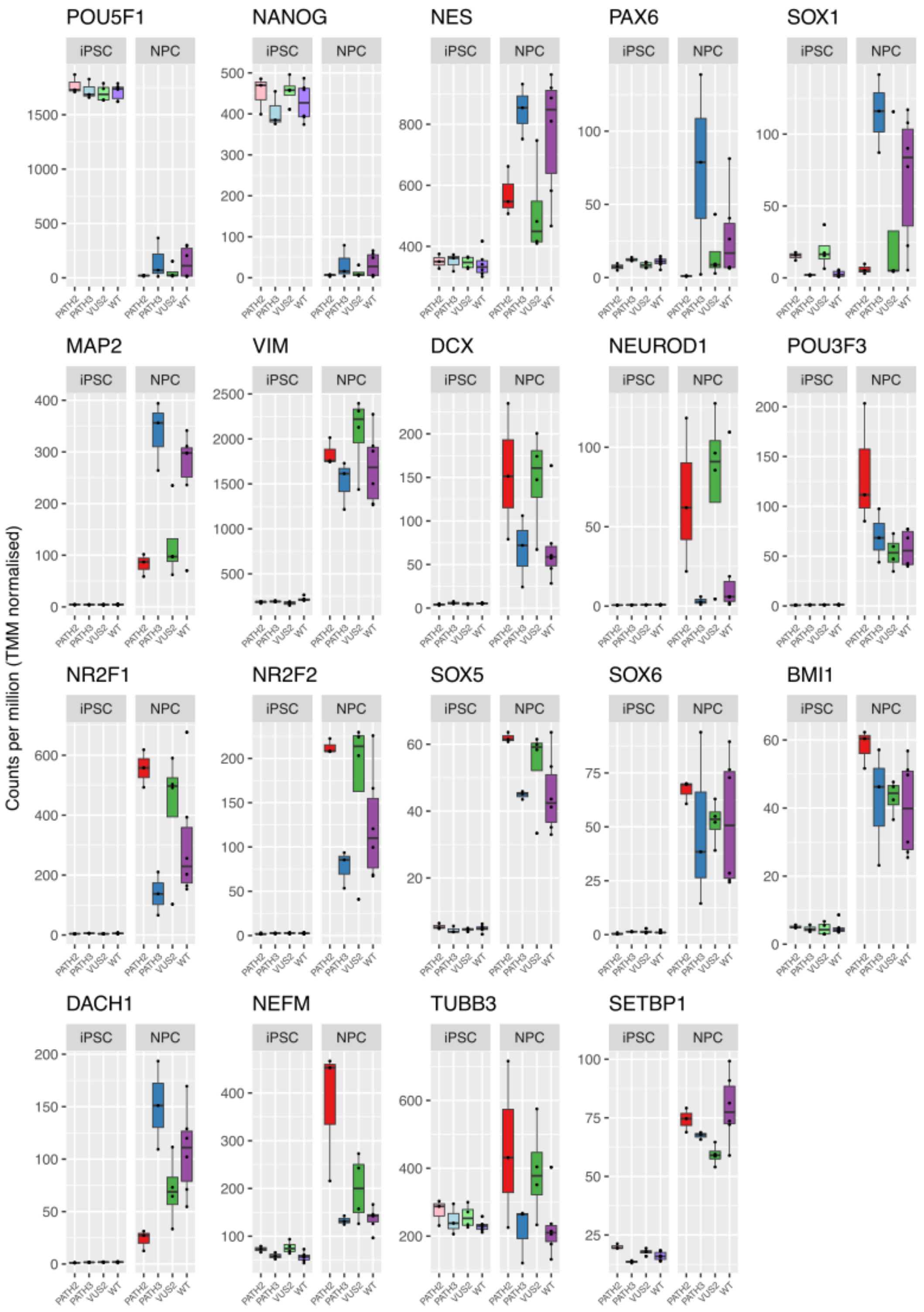

Supplementary Figure 9. Expression of selected transcripts in iPSCs and NPCs for each SETBP1 genotype. Pluripotency genes POU5F1 and NANOG transcript levels decreased over neural differentiation. Several neural associated genes increased after neural differentiation induction with some differences observed based on SETBP1 genotype. There were no significant differences in SETBP1 expression between SETBP1 variant cells and SETBP1 WT cells in iPSCs or NPCs.

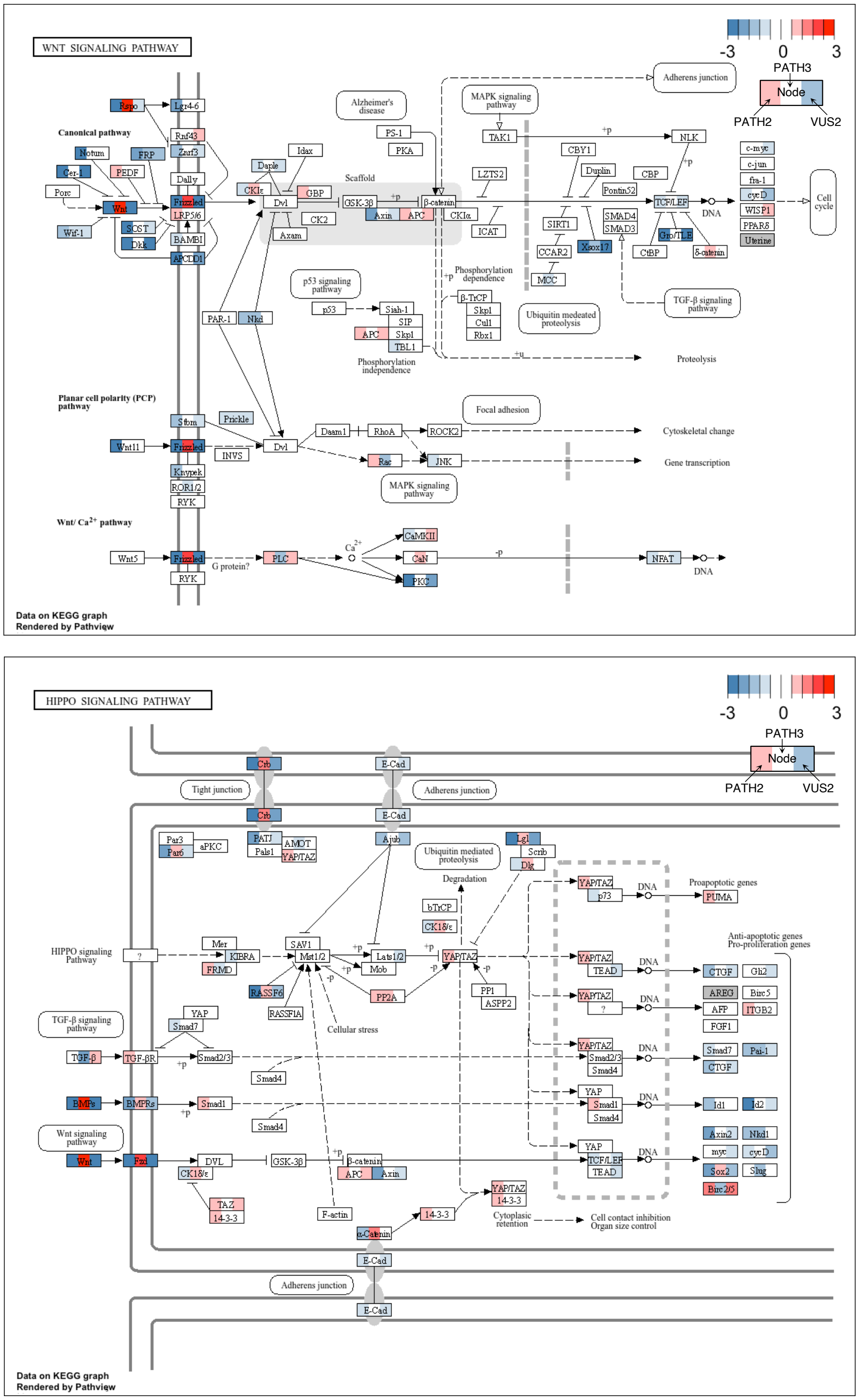

Supplementary Figure 10. Wnt and Hippo signalling pathways in SETBP1 variant NPCs. GSEA analysis of KEGG pathways for SETBP1 PATH2, PATH3 and VUS2 NPCs compared to WT NPCs revealed that the Wnt signalling pathway was significantly dysregulated for PATH2 and VUS2 NPCs and the Hippo signalling pathway was significantly dysregulated for PATH2, PATH3 and VUS2 NPCs. Log-fold changes are expressed for each pathway component as a colour gradient from blue (downregulated) to red (upregulated) for each SETBP1 variant.
